# Population resequencing of European mitochondrial genomes highlights sex-bias in Bronze Age demographic expansions

**DOI:** 10.1038/s41598-017-11307-9

**Published:** 2017-09-21

**Authors:** Chiara Batini, Pille Hallast, Åshild J. Vågene, Daniel Zadik, Heidi A. Eriksen, Horolma Pamjav, Antti Sajantila, Jon H. Wetton, Mark A. Jobling

**Affiliations:** 1 Department of Genetics & Genome Biology, University of Leicester, Leicester, UK; 2 Department of Health Sciences, University of Leicester, Leicester, UK; 3 Centre of Arctic Medicine, Thule Institute, University of Oulu, Oulu, Finland; 4 Utsjoki Health Care Centre, Utsjoki, Finland; 5 Hungarian Institute of Forensic Sciences, Institute of Forensic Genetics, Budapest, Hungary; 6 Department of Forensic Medicine, Hjelt Institute, University of Helsinki, Helsinki, Finland; 7 Institute of Applied Genetics, Department of Molecular and Medical Genetics, University of North Texas Health Science Center, Fort Worth, Texas USA; 8 Present Address: Institute of Molecular and Cell Biology, University of Tartu, Tartu, 51010 Estonia; 9 Present Address: Max Planck Institute for the Science of Human History, Jena, Germany; 10 Centre for Genetics and Genomics, University of Nottingham, Queen’s Medical Centre, Nottingham, UK

## Abstract

Interpretations of genetic data concerning the prehistory of Europe have long been a subject of great debate, but increasing amounts of ancient and modern DNA data are now providing new and more informative evidence. Y-chromosome resequencing studies in Europe have highlighted the prevalence of recent expansions of male lineages, and focused interest on the Bronze Age as a period of cultural and demographic change. These findings contrast with phylogeographic studies based on mitochondrial DNA (mtDNA), which have been interpreted as supporting expansions from glacial refugia. Here we have undertaken a population-based resequencing of complete mitochondrial genomes in Europe and the Middle East, in 340 samples from 17 populations for which Y-chromosome sequence data are also available. Demographic reconstructions show no signal of Bronze Age expansion, but evidence of Paleolithic expansions in all populations except the Saami, and with an absence of detectable geographical pattern. In agreement with previous inference from modern and ancient DNA data, the unbiased comparison between the mtDNA and Y-chromosome population datasets emphasizes the sex-biased nature of recent demographic transitions in Europe.

## Introduction

Our understanding of European prehistory has been revolutionized by the availability of new DNA sequencing technologies^1^, which have allowed the unbiased characterization of sequence variation in modern and ancient human genomes. Genome-wide ancient DNA (aDNA) data have shown a clear discontinuity between Paleolithic hunter-gatherers and Neolithic farmers^2–5^. Patterns of diversity suggested low Paleolithic population sizes, with regional differences among Western and Scandinavian groups^6^. This picture has been further refined by the study of the DNA of ancient Yamnaya herders from the region of the Pontic-Caspian steppe, the apparent source of Bronze Age migrations into Europe and Asia^7–9^, and a debated region of origin for Indo-European languages^10^. These studies highlight an introgression into Europe at around 4.5 thousand years ago (KYA) from the East, followed by development of genetic structure in Bronze Age Europe^7,8^.

Ideas on European prehistory have been strongly influenced by studies not only of autosomal DNA, but also of uniparentally-inherited markers, which can provide information about sex-biased processes^11^. Analyses of aDNA show that today’s most frequent Y-chromosome haplogroup (R1b-M269) is very rare in Europe until 4.5 KYA^5^ (see summary elsewhere^12^), while it is present in all the Yamnaya samples^8,9^. This had initially suggested a major introgression of males from the Pontic-Caspian steppe; however, the R1b sublineage (R1b-L11) now common in Europe has not so far been found among Yamnaya sequences^13^. In contrast to Y data, patterns of ancient mtDNA reveal a period of widespread turnover of lineages in the Late Glacial period ~14.5 KYA^14,15^ and support the picture of a discontinuity between Paleolithic hunter-gatherers and Neolithic farmers seen in autosomal data, but contain no apparent signal of the Bronze Age steppe expansion^16^. Ancient DNA data on uniparentally-inherited markers therefore suggest a strong sex-bias in recent demographic changes in Europe.

Resequencing of the male-specific region of the Y chromosome (MSY)^17^ in modern European populations also emphasizes the importance of Bronze-Age transition^12,18–20^. Demographic reconstructions support an expansion starting ~2.1–4.2 KYA, and times-to-most-recent-common-ancestor (TMRCAs) of three major haplogroups, including R1b, are estimated between 3.5 and 7.3 KYA^12^. Many mtDNA studies have been undertaken in modern European samples, but most concentrate on particular segments of the mitochondrial genome^21^, consider the European continent as a single unit^18,19^, or have taken a phylogeographic approach, focusing on specific lineages of interest^22–27^. General conclusions have been that current mtDNA variation represents re-expansions from glacial refugia. However, population-based whole mtDNA resequencing studies in Europe are lacking, so a systematic comparison of the demographic histories of males and females has yet not been possible.

Here, we carry out a population-based study, resequencing mtDNA in a set of 17 populations for which MSY resequencing data are already available. The pattern observed in mtDNA is strikingly different from that in MSY, compatible with expansion after the Last Glacial Maximum, and emphasizing the male-specific nature of the Bronze Age expansion in Europe.

## Results

To allow a comparison between female and male histories, we resequenced the mitochondrial genomes of 340 European and Middle Eastern individuals belonging to 17 populations (Table S1) that were previously analyzed for MSY^12^.

**Figure 1:**
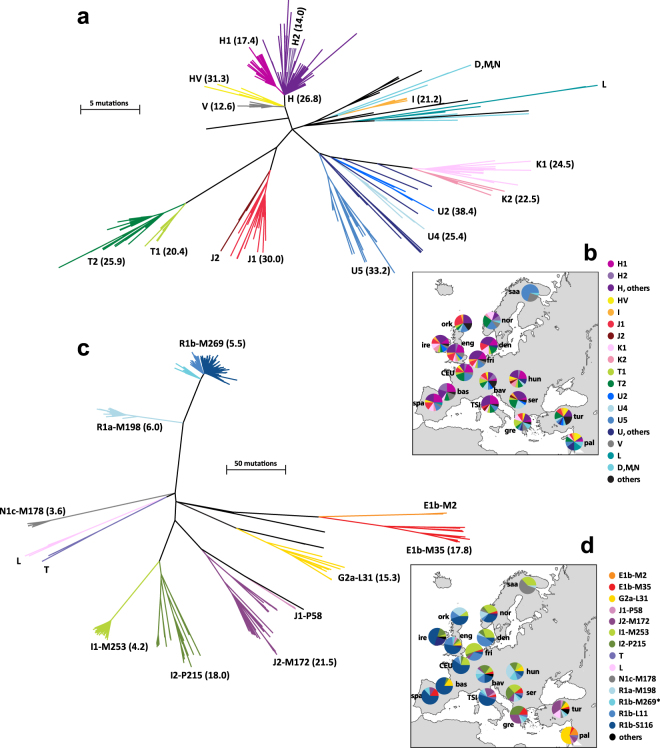
Phylogenies and geographical distributions of European mtDNA and MSY lineages. (**a**) Maximum-parsimony mtDNA tree, based on resequencing of 15,447 bp of the coding region. Branch lengths are proportional to molecular divergence among haplotypes, and colours indicate haplogroups. Point estimates of TMRCAs in KY are given in parentheses after haplogroup names; see also Table 1. (**b**) Map with pie-charts showing frequencies of mtDNA haplogroups (defined and coloured as in part (**a**)) in 17 populations from Europe and the Near East. Population abbreviations are as follows: bas: Spanish Basque country; bav: Bavaria (Germany); CEU: Utah residents with Northern and Western European ancestry from the CEPH collection (France); den: Denmark; eng: England;^41,42^ fri: Frisia (Netherlands); gre: Greece; hun: Hungary; ire: Ireland; nor: Norway; ork: Orkney;^41,42^ pal: Palestinians; saa: Saami (Finland); ser: Serbia; spa: central Spain; TSI: Toscani in Italia (Italy); tur: Turkey. Map from Mountain High Map Frontiers™ version 94.01 (Mountain High Maps® Copyright © 1993 Digital Wisdom®, Inc.; www.digiwis.com/dwi_frl.htm). (**c**) Maximum-parsimony MSY tree, based on resequencing of 3.7 Mb in each individual^12^. Branch lengths are proportional to molecular divergence among haplotypes, and colours indicate haplogroups. Point estimates of TMRCAs are given in parentheses after haplogroup names. (**d**) Map with pie-charts showing frequencies of MSY haplogroups (defined and coloured as in part (**c**)) in 17 populations from Europe and the Near East^12^. Population abbreviations are as in part (**b**). Map from Mountain High Map Frontiers™ version 94.01 (Mountain High Maps® Copyright © 1993 Digital Wisdom®, Inc.; www.digiwis.com/dwi_frl.htm).

We constructed a maximum parsimony (MP) tree (Fig. 1a; see also median-joining network in Figure S1) based on the mtDNA coding region only, which is best suited for reliable phylogenetic inference due to its relatively low content of recurrently mutating sites (Figure S2 presents a median-joining network based on the entire sequence). We also determined haplogroups from sequences (Table S1): their frequencies and geographical distributions (Fig. 1b) are broadly consistent with previous data^21^.

In the overall dataset, the main haplogroups observed are H (34.1%), U (17.9%), T (13.5%), J (9.1%), K (7.3%), and V (5.3%). The remaining 12.6% of the dataset is comprised of many minor haplogroups; these include (in the Palestinian and Spanish samples) two examples of each of the lineages L2 and L3, typically found in sub-Saharan Africa^28^ (see also YRI data in Table S1).

Visual inspection of the distributions of haplogroups (Fig. 1b) shows the Saami to be a clear outlier with low diversity, dominated by haplogroups U5 and V, and including a single example of haplogroup D, found mostly in north and east Asia^29^. In the remaining populations there is no obvious geographical pattern, in agreement with previous observations based on analyses of specific segments of the mitochondrial genome^21,30^.

This phylogeography of mitochondrial genomes was compared with that of MSY, based on resequencing of 3.7 Mb of Y-DNA in the same set of samples^12^ (Fig. 1c,d). The geographical distributions of haplogroups are more localized for MSY (Fig. 1d) than for mtDNA (Fig. 1b): for example, R1b is at particularly high frequency in the northwest, R1a in north and central Europe, and J2 in the south. Although the two MP trees have very different scales, due to the much smaller number of nucleotides analyzed in mtDNA compared to MSY (with a ratio of 1:250), both show deep-rooting clades (mtDNA: haplogroups U, K and T2; MSY: E1b-M35, G2a-L31, I2-P215, J2-M172, L and T), as well as star-like clades, indicative of population expansions. For mtDNA, these clades are H, J1, T1, V, representing 51.7% of our sample, while for MSY they are I1-M253, N1c-M178, R1a-M198 and R1b-M269 (taken together, 64%). However, the major difference is in the relative lengths of the internal branches, which indicate that the expansions of mtDNA lineages are more ancient than those of MSY lineages. This is supported by TMRCA point estimates (based on the entire mitochondrial genome) for the star-like haplogroups (Fig. 1a,c; Table 1), which are all ≤6 KYA (post-Neolithic) for the MSY^12^, but >13 KYA (Paleolithic) for mtDNA.

**Table 1:** TMRCAs of major mitochondrial haplogroups in Europe.

hg	N	TMRCA/KYA	95% HPD interval/KYA
H	116	26.8	22.5–31.9
H1	39	17.4	13.7–21.6
H2	11	14.0	7.5–22.2
HV	10	31.3	21.0–42.3
J	31	35.6	29.3–42.6
J1	28	30.0	24.2–36.0
I	8	21.2	14.8–28.6
K	25	28.8	23.3–34.4
K1	17	24.5	19.4–29.5
K2	8	22.5	16.1–29.1
T	46	30.5	25.1–36.6
T1	14	20.4	14.6–26.6
T2	32	25.9	21.2–31.0
U	61	51.1	45.5–57.1
U2	6	38.4	31.4–45.1
U4	9	25.4	18.4–33.8
U5	37	33.2	26.6–40.6
V	18	12.6	9.1–17.1

hg: haplogroup; N: number of sequences; HPD: highest posterior density; KYA: thousand years ago.

**Figure 2:**
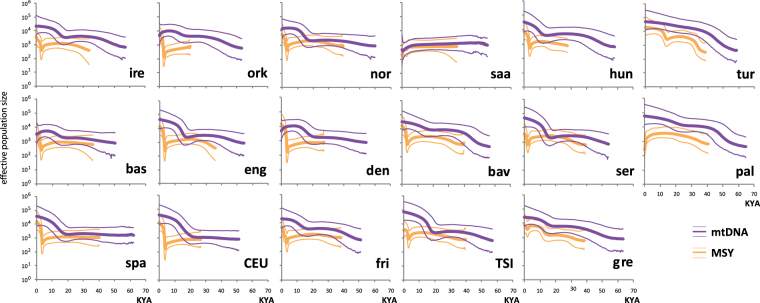
Bayesian Skyline Plots for mtDNA and MSY. Thick lines (mtDNA: purple; MSY: orange) indicate the median for effective population size and thinner lines show 95% higher posterior density intervals. Population abbreviations are as in Fig. 1, and plots for MSY are adapted from Batini *et al*. (2015)^12^.

To consider populations, rather than lineages, we reconstructed demographic histories by using Bayesian Skyline Plots (BSPs) based on mtDNA sequences (Fig. 2). As expected from their unusual haplogroup composition, the Saami also represent an outlier here, showing a steady decline in effective population size that becomes more marked at around 5 KYA. All other populations show a signature of Paleolithic expansion, between 13 and 20 KYA. The Turkish and Palestinian samples differ from the majority in showing considerably more ancient population expansion, at >40 KYA. These patterns contrast sharply with the BSPs for MSY^12^ in the same populations (Fig. 2), which in most cases (13/17 populations) show demographic histories featuring a minimum effective population size around 3 KYA (late Bronze Age for many of the populations studied), followed by rapid expansion to the present. In all comparisons except those in Basques and Danish, current point estimates of effective population size are higher for mtDNA than for MSY.

We also calculated diversity indices for each population (Table 2). In agreement with the observation of a limited number of haplogroups in Saami, and the corresponding BSP, this population shows the lowest value for all diversity measures. The highest values are seen in the Palestinian and Turkish samples, which again is concordant with the ancient population growth seen in the BSPs. We observe negative values of both *D* and *FS* for all populations except the Saami, which can indicate population growth; however, both values are significant for only twelve of the remaining populations. At a glance, there appears to be more diversity in southern than northern populations (Fig. 1b; Table 2). To formally test this, we carried out a correlation analysis between genetic diversity (number of polymorphic sites, and nucleotide diversity) and latitude, longitude and overland distance from the Franco-Cantabrian and Near-Eastern glacial refugia (Table S2). When all populations are included, both measures show a statistically significant correlation with latitude and distance from the Near-Eastern refugium, but not with longitude. These correlations are lost when we remove the outlier populations described above (Saami, Turkish and Palestinian), demonstrating the lack of any pattern of mtDNA diversity in most of Europe.

**Table 2:** Diversity parameters for the 17 populations for mtDNA coding region.

pop	*N*	k	*S*	No. usable loci	*S*/usable loci	HD (sd)	MNPD (sd)	nd (sd)	*D* (p-value)	*FS* (p-value)
bas	20	18	89	15277	0.0058	0.989 (0.019)	13.274 (6.234)	0.0009 (0.0005)	***−1.929 (0.013)***	***−5.403 (0.018)***
bav	20	20	152	15445	0.0098	1 (0.016)	23.500 (10.792)	0.0015 (0.0008)	***−1.868 (0.014)***	***−6.474 (0.010)***
CEU	20	20	105	15446	0.0068	1 (0.016)	19.047 (8.808)	0.0012 (0.0006)	−1.466 (0.063)	***−7.668 (0.003)***
den	20	17	96	15060	0.0064	0.984 (0.020)	20.268 (9.352)	0.0013 (0.0007)	−1.030 (0.133)	−2.096 (0.181)
eng	20	20	111	15446	0.0072	1 (0.016)	16.874 (7.839)	0.0011 (0.0006)	***−1.897 (0.015)***	***−8.432 (0.003)***
fri	20	19	104	15102	0.0069	0.995 (0.018)	15.905 (7.407)	0.0010 (0.0005)	***−1.881 (0.008)***	***−6.263 (0.011)***
gre	20	20	148	15044	0.0098	1 (0.016)	22.556 (10.372)	0.0015 (0.0008)	***−1.900 (0.017)***	***−6.694 (0.009)***
hun	20	20	123	15256	0.0081	1 (0.016)	19.374 (8.953)	0.0013 (0.0006)	***−1.820 (0.025)***	***−7.566 (0.007)***
ire	20	19	114	14940	0.0076	0.995 (0.018)	19.821 (9.152)	0.0013 (0.0007)	***−1.578 (0.040)***	***−5.064 (0.025)***
nor	20	18	123	15308	0.0080	0.989 (0.019)	23.647 (10.857)	0.0015 (0.0008)	−1.311 (0.073)	−2.712 (0.116)
ork	20	17	114	15446	0.0074	0.984 (0.020)	20.453 (9.434)	0.0013 (0.0007)	−1.497 (0.056)	−2.064 (0.181)
pal	20	20	201	15446	0.0130	1 (0.016)	27.784 (12.700)	0.0018 (0.0009)	***−2.116 (0.006)***	***−5.634 (0.012)***
saa	20	7	42	15160	0.0028	0.737 (0.094)	10.132 (4.832)	0.0007 (0.0004)	−0.576 (0.278)	4.841 (0.955)
ser	20	20	116	15026	0.0077	1 (0.016)	20.721 (9.553)	0.0014 (0.0007)	***−1.509 (0.050)***	***−7.170 (0.003)***
spa	20	20	124	15393	0.0081	1 (0.016)	19.400 (8.965)	0.0013 (0.0006)	***−1.835 (0.018)***	***−7.558 (0.003)***
TSI	20	20	131	15446	0.0085	1 (0.016)	19.874 (9.176)	0.0013 (0.0007)	***−1.907 (0.022)***	***−7.414 (0.005)***
tur	20	20	172	15401	0.0112	1 (0.016)	24.237 (11.120)	0.0016 (0.0008)	***−2.073 (0.009)***	***−6.312 (0.010)***

pop: population; *N*: number of individuals; k: number of haplotypes; *S*: number of polymorphic sites; HD: haplotype diversity; sd: standard deviation; MNPD: mean number of pairwise differences; nd: nucleotide diversity; *D*: Tajima’s *D* (bold italic values are significant); *FS*: Fu’s FS (bold italic values are significant).

## Discussion

The study of mtDNA in Europe has a long history and has been influential in developing hypotheses about the origins of modern Europeans^15,31^. Early population-based studies involved sequencing of the control region^32^, and later the typing of specific haplogroup-defining SNPs in the coding region^21^. Analyses of whole mitochondrial genome sequences have focused on specific haplogroups, often in the framework of the re-peopling of Europe after the last glacial maximum^23–25^. At the population scale, the only previous studies have considered Europe at the continental level^18,19^, and with low sample sizes (n = 86 and 81 respectively), and patchy population coverage.

Here, we have carried out whole mtDNA sequencing in a European and Middle Eastern population-based sample set of 340 individuals, in which MSY resequencing^12^ had previously been undertaken. The spectrum of haplogroups we observe (Table S1, Fig. 1a) is compatible with previously published data^21^. The population-based design of this study and the unbiased nature of variant ascertainment means that European mtDNA and MSY diversity can be compared fairly. The phylogenies and demographic reconstructions concur in showing a marked difference between female and male population histories, with Paleolithic expansions for mtDNA contrasting with Bronze Age expansions for MSY. While this is in agreement with continental-level differences observed previously^18,19^, here we also show that this difference holds for most of the individual populations, and reflects a lack of geographical pattern in Europe. The most ancient mtDNA expansions we detect, dating close to the early peopling of Eurasia (40–50 KYA), are in the Near and Middle East. This difference in timing of European female and male lineage expansions is mirrored in the Indian subcontinent, where a recent analysis^33^ shows that mtDNA expansions reflect processes in the pre-Holocene era, while MSY expansions are mostly in the last 10 KYA, with marked male-driven spread from Central Asia during the Bronze Age.

Since mtDNA analysis has forensic utility^34^, it is worth noting that the 340 individuals carry only 318 distinct haplotypes based on complete mitochondrial genomes (Figure S2; Table S3), emphasizing the relatively low discriminatory power of mtDNA sequencing, even at its maximum resolution. We observe 12 identical pairs of haplotypes, three trios, and one example of a haplotype found five times in the dataset. These cases represent within-population sharing (in Danish, Norwegian, Orcadian, Frisian, Basque and Saami), with one exception, a haplotype within hg V shared between two Saami and a Spanish individual (Figure S2b) – reflecting a connection previously noted^35^. As reflected in diversity measures (Table 2) the Saami have particularly high haplotype sharing (two pairs, two trios, one quintet). These findings emphasize the importance of large and appropriate reference databases in forensic analysis.

The outlier status of the Saami in our dataset of 17 populations is clear not only from the high frequencies of closely related mtDNA sequences (Figure S2), but also haplogroups for both mtDNA and MSY that are rare elsewhere in the dataset (Fig. 1b,d), as well as examples of MSY sequences^36^ and Y-STR haplotypes^36^ that are found in more than one individual. These features are in agreement with the lack of growth in effective population size seen in the BSP (Fig. 2). Population-based genome-wide SNP analysis^37^ and whole-genome sequencing of a single individual^38^ also show the Saami to be genetically differentiated compared to Europeans, and to carry East Asian ancestry components.

Our data are consistent with ancient DNA data^14–16^ in supporting sex-biased processes in recent European demographic changes: patterns of modern mtDNA diversity show no signal of the Bronze Age expansion, while much of the modern European MSY diversity has been shaped by this process^12^. However, the modern data differ in showing no clear signal of the Neolithic transition that has been highlighted in ancient mitochondrial and autosomal data^5,16^. This could be due to drift, which is important in shaping the observed patterns of diversity in uniparental markers, and also sampling effects.

Much progress has been made in understanding the prehistory of the European continent since the first classical genetic data were interpreted in favour of agriculturally-mediated demic diffusion^39^. A wealth of both modern and ancient DNA data is now available, and has highlighted previously unsuspected past migration and expansion events, with a sex-biased aspect supported by our population-based resequencing approach. However, there is still much future work to be done in increasing sample sizes and geographical coverage, and in fully integrating the ancient and modern data to test explicitly the complex scenarios they suggest.

## Materials and Methods

### DNA samples and sequencing

DNA samples from 340 individuals belonging to 17 populations (20 individuals each) across Europe and the Near East were used for analysis, as in our previous study of MSY diversity^12^. Populations were as follows: Greek, Serbian, Hungarian, German [Bavaria], Spanish Basque, central Spanish, French (Centre d’Etude du Polymorphisme Humain [CEPH] collection in Utah, USA, with ancestry from Northern and Western Europe^40^ [CEU]), Italian (Toscani in Italia^40^ [TSI]), Dutch [Frisia], Danish, Norwegian, Finnish [Saami], English^41,42^ [Herefordshire and Worcestershire, Gloucestershire, Oxfordshire, Forest of Dean], Orcadian^41,42^, Irish, Turkish and Palestinian. Twenty individuals from each of two additional HapMap^40^ population samples, CHB (Han Chinese in Beijing, China), and YRI (Yoruba in Ibadan, Nigeria) were included to provide variant validation data. All methods were carried out in accordance with relevant guidelines and regulations, and all experimental protocols were approved by the University of Leicester Research Ethics Committee. Informed consent was obtained from all subjects (University of Leicester Research Ethics Committee reference: maj4-cb66).

We generated three datasets using parallel sequencing strategies (based on Illumina HiSeq, Illumina MiSeq and Ion Torrent PGM technologies [Table S4]) and bioinformatic pipelines (Table S5), and validated variants by comparison with independent sequence and SNP-genotype data (Table S6). These three datasets were merged for all the subsequent evolutionary analyses. The 380 sequences are available in Supplementary Dataset S1.

### Tree construction and haplogroup prediction

A maximum parsimony (MP) tree was constructed from coding-region sequences (positions 576-16,023) via MEGA6^43^, using the Subtree-Pruning-Regrafting (SPR) algorithm^44^ with search level 0 in which the initial trees were obtained by the random addition of sequences (10 replicates). Branch lengths were calculated using the average pathway method^44^ and are proportional to the number of mutations. FigTree v1.4.0^45^ was used for tree visualization. Haplogroups were predicted using HaploGrep2^46^, and their phylogenetic coherence was verified using the tree, with manual examination of possible ‘phantom’ mutations, as inferred using Haplogrep2^46^ (Table S7).

Median-joining networks^47^ based on either coding region or whole mtDNA sequences were constructed using Network 5.0.0.0, and represented using Network Publisher 2.1.1.2. Polymorphic sites were weighted according to their evolutionary rates using the parameters suggested in the literature^48^.

Haplogroups were defined according to Phylotree16^49^ by using HaploGrep^50^ and their relative frequencies represented as pie-charts plotted on a geographical map.

### TMRCA estimation

TMRCAs of nodes of interest were estimated^51^ using BEAST v1.8.0. MCMC samples were based on 50,000,000 generations, logging every 1000 steps, with the first 5,000,000 generations discarded as burn-in. Three runs were combined for analysis using LogCombiner. We used an exponential growth coalescent tree prior, HKY substitution model, and an uncorrelated relaxed clock with a lognormal distribution for mutation rate (2.21 ± 0.17 × 10^−8^ mutations/nucleotide/year^52^). TMRCAs were estimated in a single run including all 17 populations and assigning samples to specific clades in agreement with the MP tree shown in Fig. 1. For this analysis, the entire mitochondrial genome was considered: given the timeframe of interest (<40 KYA), the rate and its standard error were adjusted by using the calculator^52^ in Soares *et al*. (2009) to infer the rate at each of the nodes of interest (see Table 1). The median of this distribution of values was used for estimating TMRCAs for the haplogroups of interest.

### Bayesian skyline plots

BSPs^51^ were generated using BEAST v1.8.0. MCMC samples were based on 30,000,000 generations, logging every 1000 steps, with the first 3,000,000 generations discarded as burn-in. We used a piecewise linear skyline model with ten groups, a HKY substitution model, and an uncorrelated relaxed clock with a lognormal distribution for mutation rate (2.21 ± 0.17 × 10^−8^ mutations/nucleotide/year^52^) and a generation time of 30 years^53,54^. For this analysis, the entire mitochondrial genome was considered: given the timeframe of interest (<40 KYA) the rate and its standard error were adjusted by using the calculator^52^ in Soares *et al*. (2009) to infer the rate at each of the nodes of interest (see Table 1). The median of this distribution of values was used for estimating TMRCAs for the haplogroups of interest.

### Intrapopulation diversity and geographical correlation

The number of polymorphic sites per population (*S*), nucleotide diversity, Tajima’s *D* ^55^, and Fu’s *FS* ^56^ were calculated^57^ using Arlequin 3.5. Correlations of *S*, and of nucleotide diversity, with latitude, longitude, and distances from glacial refugia were examined using the function cor.test of the package stats within R. The locations Anamur, Turkey (36.1°, 32.8°) and Fleurac, France (45.0°, 1.0°) were taken as proxies for the centres of the Near-Eastern and Franco-Cantabrian refugia respectively. Distances account for geographical barriers, and were estimated using the land transport distance tool at www.freemaptools.com.

### Data availability

All data generated during this study are included in this article (and its Supplementary Information files).

## Electronic supplementary material

Supplementary Information

Supplementary Dataset S1
