# Supplementary material for: Population resequencing of European mitochondrial genomes highlights sex-bias in Bronze Age demographic expansions
Source: Sci Rep. 2017 Sep 21;7:12086. doi: 10.1038/s41598-017-11307-9 (PMC5608872; doi:10.1038/s41598-017-11307-9)
Supplement: Supplementary file 1 — Supplementary Information [file 41598_2017_11307_MOESM1_ESM.pdf]

# Supplementary Information for:

## Population resequencing of European mitochondrial genomes highlights sex-bias in Bronze Age demographic expansions

Chiara Batini, Pille Hallast, Åshild J. Vågene, Daniel Zadik, Heidi A. Eriksen, Horolma Pamjav, Antti Sajantila, Jon H. Wetton, Mark A. Jobling

***Contents:***

Figure S1: Median-joining network of mtDNA haplotypes based on coding region variants.

Figure S2: Median-joining network of mtDNA haplotypes based on all variants.

Figure S3: Bayesian skyline plots for the YRI and CHB population samples.

Table S1: Sample information.

Table S2: Correlations of genetic diversity measures with latitude, longitude and distances from glacial refugia.

Table S3: Diversity parameters for the 17 populations for complete mtDNA sequences.

Table S4: DNA sequencing information.

Table S5: Software tools and parameters used in NGS data analysis.

Table S6: Variant validation summary.

Table S7: List of possible 'phantom' mutations.

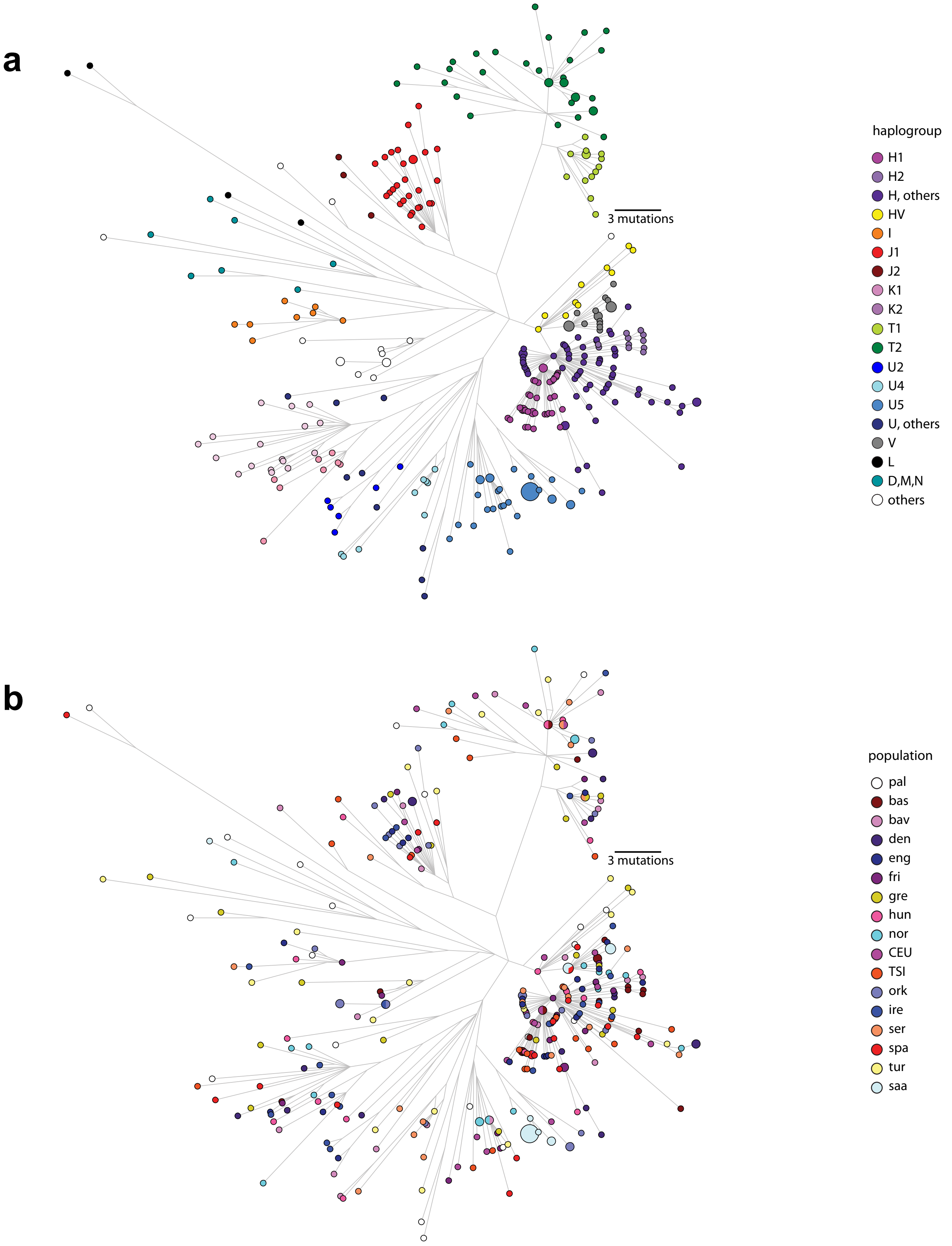


**Figure S1: Median-joining network of mtDNA haplotypes based on coding region variants.**
Circles represent haplotypes, with area proportional to frequency, and are coloured according to:

a) Haplogroup, as indicated in the key.

b) Population, as indicated in the key, with population abbreviations as given in Figure 1.

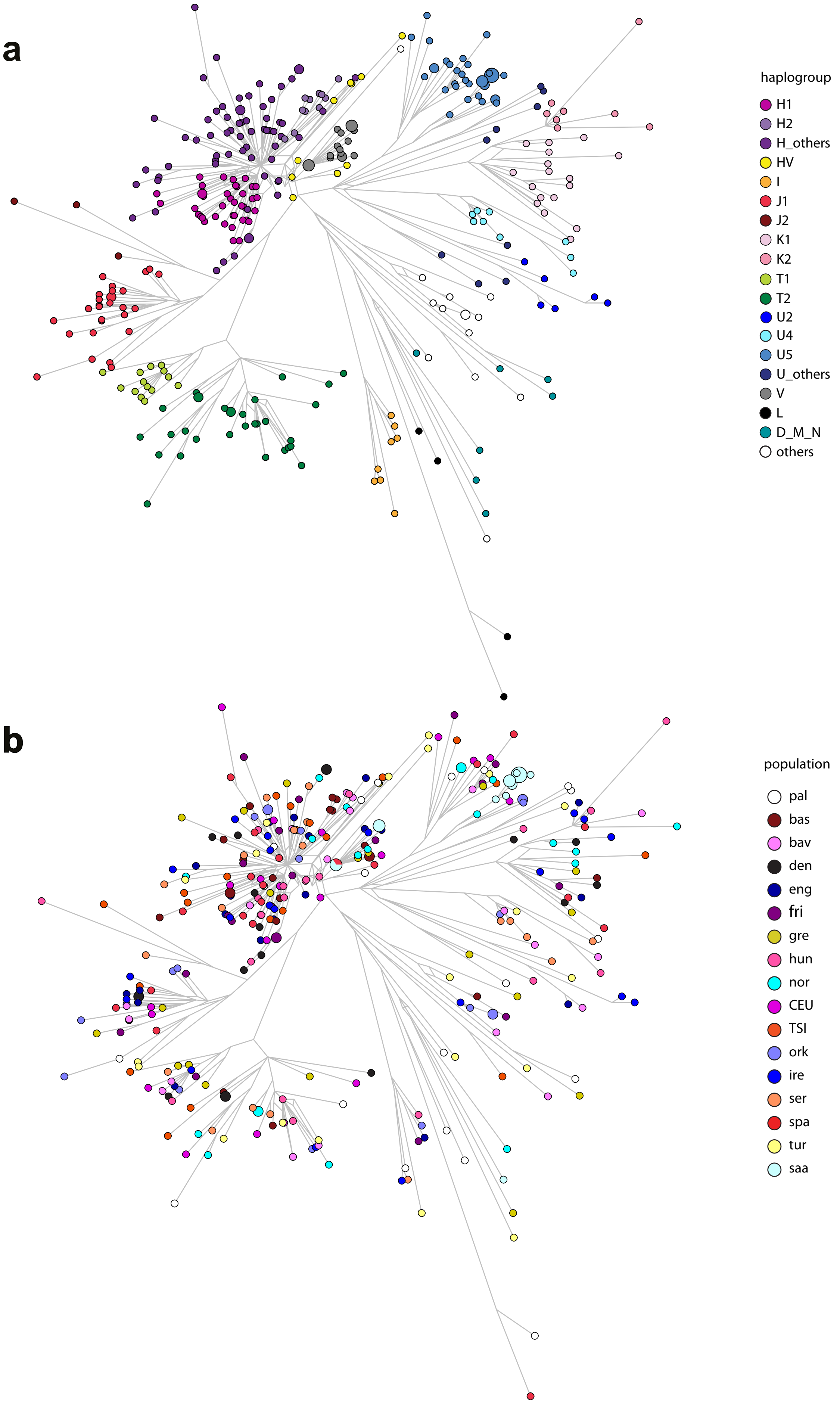


**Figure S2: Median-joining network of mtDNA haplotypes based on all variants.**
Circles represent haplotypes, with area proportional to frequency, and are coloured according to:

a) Haplogroup, as indicated in the key.

b) Population, as indicated in the key, with population abbreviations as given in Figure 1.

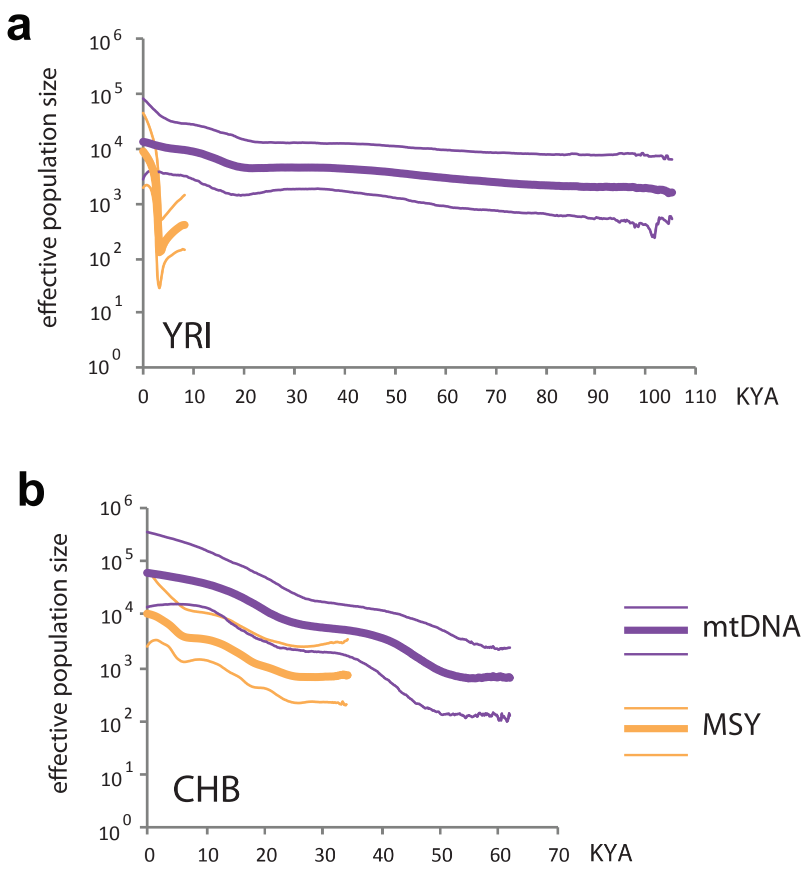


**Figure S3: Bayesian skyline plots for the YRI and CHB population samples.**
Thick lines (mtDNA: purple; MSY: orange) indicate the median for effective population size and thinner lines show 95% higher posterior density intervals.

**Table S1: Sample information.**

| Sample name | Population | Sequencing platform | Mean coverage (BQ20, MQ50) | Main filters | Sites with missing data | Number of variants compared to rCRS | % missing data | Haplogroup | HaploGrep prediction quality (%) |
|---|---|---|---|---|---|---|---|---|---|
| bas-1 | Basque | IonTorrent | 72 | BQ20, MQ50, DP2 | 1 | 13 | 0.000 | H53 | 87.4 |
| bas-10 | Basque | IonTorrent | 70 | BQ20, MQ50, DP2 | 7 | 9 | 0.000 | H2a5a1 | 92.9 |
| bas-11 | Basque | IonTorrent | 36 | BQ20, MQ50, DP2 | 243 | 10 | 0.015 | H1t1 | 100 |
| bas-12 | Basque | Illumina MiSeq | 2447 | BQ20, MQ50, DP2 | 0 | 13 | 0.000 | H1 | 82.7 |
| bas-13 | Basque | Illumina MiSeq | 2270 | BQ20, MQ50, DP2 | 0 | 12 | 0.000 | H3c2a | 99 |
| bas-14 | Basque | Illumina MiSeq | 2352 | BQ20, MQ50, DP2 | 0 | 34 | 0.000 | T2b | 95.1 |
| bas-15 | Basque | Illumina HiSeq | 15 | BQ20, MQ50, DP1 | 580 | 10 | 0.035 | H17a | 92.1 |
| bas-16 | Basque | IonTorrent | 65 | BQ20, MQ50, DP2 | 3 | 33 | 0.000 | T2e | 99.6 |
| bas-17 | Basque | Illumina HiSeq | 27 | BQ20, MQ50, DP1 | 30 | 30 | 0.002 | X2c1 | 100 |
| bas-18 | Basque | Illumina HiSeq | 25 | BQ20, MQ50, DP1 | 51 | 18 | 0.003 | H4a1a1a1a | 100 |
| bas-19 | Basque | Illumina HiSeq | 15 | BQ20, MQ50, DP1 | 170 | 9 | 0.010 | H1 | 97.9 |
| bas-2 | Basque | Illumina HiSeq | 14 | BQ20, MQ50, DP1 | 534 | 13 | 0.032 | V | 79.6 |
| bas-20 | Basque | IonTorrent | 55 | BQ20, MQ50, DP2 | 5 | 15 | 0.000 | V22 | 100 |
| bas-3 | Basque | Illumina MiSeq | 2252 | BQ20, MQ50, DP2 | 0 | 13 | 0.000 | H1 | 82.7 |
| bas-4 | Basque | Illumina MiSeq | 1738 | BQ20, MQ50, DP2 | 0 | 34 | 0.000 | K1a4a1e | 98.8 |
| bas-5 | Basque | Illumina MiSeq | 2830 | BQ20, MQ50, DP2 | 0 | 10 | 0.000 | H2a5a1a | 94.7 |
| bas-6 | Basque | Illumina MiSeq | 1623 | BQ20, MQ50, DP2 | 0 | 9 | 0.000 | H2a5a1a | 100 |
| bas-7 | Basque | Illumina MiSeq | 1660 | BQ20, MQ50, DP2 | 0 | 10 | 0.000 | H2a5a1a | 94.1 |
| bas-8 | Basque | Illumina MiSeq | 1613 | BQ20, MQ50, DP2 | 0 | 15 | 0.000 | V22 | 100 |
| bas-9 | Basque | Illumina MiSeq | 2333 | BQ20, MQ50, DP2 | 0 | 12 | 0.000 | H1t1a | 97.1 |
| bav-12 | Bavarian | IonTorrent | 38 | BQ20, MQ50, DP2 | 8 | 12 | 0.000 | H1a1 | 100 |
| bav-13 | Bavarian | Illumina MiSeq | 2561 | BQ20, MQ50, DP2 | 0 | 31 | 0.000 | U7b | 94 |
| bav-19 | Bavarian | IonTorrent | 65 | BQ20, MQ50, DP2 | 5 | 34 | 0.000 | U4c1a | 95 |
| bav-26 | Bavarian | Illumina MiSeq | 1569 | BQ20, MQ50, DP2 | 0 | 14 | 0.000 | H | 79.2 |
| bav-27 | Bavarian | Illumina MiSeq | 1515 | BQ20, MQ50, DP2 | 0 | 10 | 0.000 | H7 | 91.7 |
| bav-29 | Bavarian | Illumina MiSeq | 3678 | BQ20, MQ50, DP2 | 0 | 37 | 0.000 | T2b1 | 98.3 |
| bav-30 | Bavarian | Illumina MiSeq | 2250 | BQ20, MQ50, DP2 | 0 | 2 | 0.000 | H2a2a1a | 73.2 |
| bav-31 | Bavarian | Illumina MiSeq | 2183 | BQ20, MQ50, DP2 | 0 | 33 | 0.000 | X2b | 93.8 |
| bav-32 | Bavarian | IonTorrent | 76 | BQ20, MQ50, DP2 | 11 | 36 | 0.001 | T2b | 95.2 |
| bav-38 | Bavarian | Illumina MiSeq | 1957 | BQ20, MQ50, DP2 | 0 | 36 | 0.000 | K2a5 | 93.6 |
| bav-39 | Bavarian | Illumina MiSeq | 1914 | BQ20, MQ50, DP2 | 0 | 36 | 0.000 | T1a1 | 97.5 |
| bav-40 | Bavarian | Illumina MiSeq | 2208 | BQ20, MQ50, DP2 | 0 | 12 | 0.000 | HV0 | 91.8 |
| bav-41 | Bavarian | Illumina MiSeq | 1911 | BQ20, MQ50, DP2 | 0 | 28 | 0.000 | U4a2 | 98.6 |
| bav-52 | Bavarian | Illumina MiSeq | 3027 | BQ20, MQ50, DP2 | 0 | 35 | 0.000 | T1a1 | 96.4 |
| bav-53 | Bavarian | Illumina MiSeq | 2766 | BQ20, MQ50, DP2 | 0 | 25 | 0.000 | U5a1a1 | 100 |
| bav-54 | Bavarian | Illumina MiSeq | 4334 | BQ20, MQ50, DP2 | 0 | 33 | 0.000 | J1c2q | 93.3 |
| bav-55 | Bavarian | IonTorrent | 78 | BQ20, MQ50, DP2 | 7 | 33 | 0.000 | J1c2e1 | 98 |
| bav-56 | Bavarian | Illumina MiSeq | 2390 | BQ20, MQ50, DP2 | 0 | 38 | 0.000 | W5a1a1 | 100 |
| bav-57 | Bavarian | Illumina MiSeq | 2018 | BQ20, MQ50, DP2 | 0 | 38 | 0.000 | U2e1a1 | 97 |
| bav-8 | Bavarian | IonTorrent | 43 | BQ20, MQ50, DP2 | 13 | 2 | 0.001 | H2a2a1 | 50 |
| den-104 | Danish | Illumina MiSeq | 3295 | BQ20, MQ50, DP2 | 0 | 11 | 0.000 | H1a | 94.2 |
| den-113 | Danish | Illumina MiSeq | 2080 | BQ20, MQ50, DP2 | 0 | 12 | 0.000 | H3h1 | 94.6 |
| den-150 | Danish | Illumina HiSeq | 20 | BQ20, MQ50, DP1 | 84 | 31 | 0.005 | K1a | 91.2 |
| den-152 | Danish | Illumina MiSeq | 1827 | BQ20, MQ50, DP2 | 0 | 15 | 0.000 | H13a1a1a | 96.1 |
| den-157 | Danish | Illumina HiSeq | 20 | BQ20, MQ50, DP1 | 316 | 32 | 0.019 | J1c5a1 | 96.9 |
| den-158 | Danish | Illumina HiSeq | 22 | BQ20, MQ50, DP1 | 136 | 37 | 0.008 | T2e1a | 92 |
| den-176 | Danish | IonTorrent | 102 | BQ20, MQ50, DP2 | 7 | 16 | 0.000 | H11a | 89.6 |
| den-183 | Danish | Illumina HiSeq | 21 | BQ20, MQ50, DP1 | 56 | 15 | 0.003 | H11a | 91.5 |
| den-186 | Danish | Illumina HiSeq | 21 | BQ20, MQ50, DP1 | 156 | 28 | 0.009 | U8a1b | 94.4 |
| den-189 | Danish | Illumina HiSeq | 20 | BQ20, MQ50, DP1 | 300 | 14 | 0.018 | H13a1a1a | 93.6 |
| den-190 | Danish | IonTorrent | 66 | BQ20, MQ50, DP2 | 3 | 13 | 0.000 | H10e | 89.5 |
| den-191 | Danish | Illumina HiSeq | 22 | BQ20, MQ50, DP1 | 82 | 36 | 0.005 | K1a4a1 | 95.4 |
| den-192 | Danish | Illumina HiSeq | 23 | BQ20, MQ50, DP1 | 33 | 32 | 0.002 | J1c4 | 96.4 |
| den-207 | Danish | Illumina HiSeq | 26 | BQ20, MQ50, DP1 | 72 | 13 | 0.004 | H1c1 | 91.2 |
| den-30 | Danish | IonTorrent | 95 | BQ20, MQ50, DP2 | 1 | 36 | 0.000 | T1a1k | 96.4 |
| den-34 | Danish | IonTorrent | 57 | BQ20, MQ50, DP2 | 4 | 32 | 0.000 | J1c4 | 96.4 |
| den-36 | Danish | Illumina MiSeq | 3354 | BQ20, MQ50, DP2 | 0 | 34 | 0.000 | K1c1b | 96.6 |
| den-50-51 | Danish | Illumina MiSeq | 3075 | BQ20, MQ50, DP2 | 0 | 35 | 0.000 | T2 | 95 |
| den-82-83 | Danish | Illumina MiSeq | 2287 | BQ20, MQ50, DP2 | 0 | 35 | 0.000 | T2 | 95 |
| den-SN | Danish | Illumina HiSeq | 25 | BQ20, MQ50, DP1 | 76 | 14 | 0.005 | H1c3b | 100 |
| eng-F2 | English | Illumina HiSeq | 70 | BQ20, MQ50, DP1 | 10 | 32 | 0.001 | K2a | 96.7 |
| eng-F58 | English | Illumina HiSeq | 58 | BQ20, MQ50, DP1 | 8 | 37 | 0.000 | U2e1a1 | 98.7 |
| eng-F6 | English | IonTorrent | 98 | BQ20, MQ50, DP2 | 1 | 35 | 0.000 | K1a4a1b | 96.8 |
| eng-G9 | English | Illumina HiSeq | 71 | BQ20, MQ50, DP1 | 3 | 14 | 0.000 | H1k1 | 95.8 |
| eng-H18 | English | Illumina HiSeq | 42 | BQ20, MQ50, DP1 | 46 | 16 | 0.003 | V2 | 92 |
| eng-H38 | English | IonTorrent | 59 | BQ20, MQ50, DP2 | 2 | 15 | 0.000 | H17c | 93.2 |

| | | | | | | | | | |
|---|---|---|---|---|---|---|---|---|---|
| eng-N5 | English | Illumina HiSeq | 162 | BQ20, MQ50, DP1 | 2 | 29 | 0.000 | J1c1b | 96.6 |
| eng-O109 | English | Illumina HiSeq | 86 | BQ20, MQ50, DP1 | 2 | 10 | 0.000 | H | 92.6 |
| eng-O15 | English | Illumina HiSeq | 75 | BQ20, MQ50, DP1 | 3 | 14 | 0.000 | H1a5 | 99.5 |
| eng-O23 | English | Illumina HiSeq | 133 | BQ20, MQ50, DP1 | 0 | 17 | 0.000 | V | 94.3 |
| eng-O36 | English | Illumina HiSeq | 167 | BQ20, MQ50, DP1 | 0 | 13 | 0.000 | H1o | 92.5 |
| eng-O44 | English | Illumina HiSeq | 80 | BQ20, MQ50, DP1 | 5 | 11 | 0.000 | H7 | 98 |
| eng-O45 | English | Illumina HiSeq | 128 | BQ20, MQ50, DP1 | 3 | 35 | 0.000 | T1a1 | 96.8 |
| eng-O63 | English | Illumina HiSeq | 68 | BQ20, MQ50, DP1 | 3 | 30 | 0.000 | J1c3a1 | 95.4 |
| eng-O69 | English | Illumina HiSeq | 102 | BQ20, MQ50, DP1 | 2 | 10 | 0.000 | H5a1 | 100 |
| eng-O93 | English | Illumina HiSeq | 72 | BQ20, MQ50, DP1 | 1 | 35 | 0.000 | I2 | 95.7 |
| eng-W11 | English | IonTorrent | 75 | BQ20, MQ50, DP2 | 3 | 29 | 0.000 | J1c3a1 | 98.5 |
| eng-W13 | English | Illumina HiSeq | 86 | BQ20, MQ50, DP1 | 3 | 4 | 0.000 | H2a2a1 | 50 |
| eng-W17 | English | Illumina HiSeq | 58 | BQ20, MQ50, DP1 | 4 | 10 | 0.000 | H24b | 95.4 |
| eng-W20 | English | Illumina HiSeq | 51 | BQ20, MQ50, DP1 | 2 | 15 | 0.000 | H1b1 | 88.7 |
| CEU-NA06994 | French (CEU) | Illumina HiSeq | 83 | BQ20, MQ50, DP1 | 2 | 27 | 0.000 | U5b2a1a1 | 100 |
| CEU-NA07048 | French (CEU) | Illumina HiSeq | 94 | BQ20, MQ50, DP1 | 1 | 6 | 0.000 | H2a1 | 100 |
| CEU-NA07357 | French (CEU) | Illumina HiSeq | 95 | BQ20, MQ50, DP1 | 4 | 30 | 0.000 | U5a1c2a | 92.9 |
| CEU-NA10851 | French (CEU) | Illumina HiSeq | 108 | BQ20, MQ50, DP1 | 1 | 16 | 0.000 | V16 | 96.8 |
| CEU-NA11829 | French (CEU) | Illumina HiSeq | 100 | BQ20, MQ50, DP1 | 3 | 39 | 0.000 | T2a1a | 94 |
| CEU-NA11831 | French (CEU) | Illumina HiSeq | 81 | BQ20, MQ50, DP1 | 3 | 9 | 0.000 | H5'36 | 91.5 |
| CEU-NA11992 | French (CEU) | Illumina HiSeq | 102 | BQ20, MQ50, DP1 | 0 | 28 | 0.000 | U5a2d1a | 100 |
| CEU-NA11994 | French (CEU) | Illumina HiSeq | 132 | BQ20, MQ50, DP1 | 0 | 35 | 0.000 | T2b4b | 97.2 |
| CEU-NA12003 | French (CEU) | Illumina HiSeq | 84 | BQ20, MQ50, DP1 | 2 | 34 | 0.000 | T2b1 | 98.3 |
| CEU-NA12043 | French (CEU) | Illumina HiSeq | 82 | BQ20, MQ50, DP1 | 4 | 34 | 0.000 | T1 | 96.4 |
| CEU-NA12144 | French (CEU) | Illumina HiSeq | 81 | BQ20, MQ50, DP1 | 5 | 13 | 0.000 | H1e1a | 93.7 |
| CEU-NA12154 | French (CEU) | Illumina HiSeq | 95 | BQ20, MQ50, DP1 | 1 | 30 | 0.000 | J1c5a1 | 100 |
| CEU-NA12155 | French (CEU) | Illumina HiSeq | 71 | BQ20, MQ50, DP1 | 4 | 20 | 0.000 | H8c | 86 |
| CEU-NA12707 | French (CEU) | Illumina HiSeq | 109 | BQ20, MQ50, DP1 | 2 | 11 | 0.000 | H1b1 | 96.5 |
| CEU-NA12716 | French (CEU) | Illumina HiSeq | 206 | BQ20, MQ50, DP1 | 0 | 14 | 0.000 | H1ae2a | 91.4 |
| CEU-NA12750 | French (CEU) | Illumina HiSeq | 83 | BQ20, MQ50, DP1 | 12 | 39 | 0.001 | T2f1a1 | 88.6 |
| CEU-NA12812 | French (CEU) | Illumina HiSeq | 109 | BQ20, MQ50, DP1 | 2 | 12 | 0.000 | H1 | 94.3 |
| CEU-NA12814 | French (CEU) | Illumina HiSeq | 83 | BQ20, MQ50, DP1 | 2 | 29 | 0.000 | U5a1b1a2 | 98.7 |
| CEU-NA12872 | French (CEU) | Illumina HiSeq | 72 | BQ20, MQ50, DP1 | 3 | 11 | 0.000 | HV6 | 100 |
| CEU-NA12874 | French (CEU) | Illumina HiSeq | 75 | BQ20, MQ50, DP1 | 4 | 28 | 0.000 | J1c8a | 98.4 |
| fri-1048 | Frisian | IonTorrent | 73 | BQ20, MQ50, DP2 | 3 | 11 | 0.000 | H17 | 93.5 |
| fri-1087 | Frisian | Illumina HiSeq | 27 | BQ20, MQ50, DP1 | 66 | 14 | 0.004 | H1a1 | 96.5 |
| fri-1309 | Frisian | Illumina HiSeq | 44 | BQ20, MQ50, DP1 | 25 | 31 | 0.002 | X2c1 | 94.3 |
| fri-1311 | Frisian | IonTorrent | 53 | BQ20, MQ50, DP2 | 1 | 10 | 0.000 | H | 86.8 |
| fri-1312 | Frisian | Illumina HiSeq | 42 | BQ20, MQ50, DP1 | 28 | 11 | 0.002 | H1c2a | 100 |
| fri-1313 | Frisian | Illumina MiSeq | 3568 | BQ20, MQ50, DP2 | 0 | 28 | 0.000 | J1c5 | 96 |
| fri-1317 | Frisian | Illumina HiSeq | 20 | BQ20, MQ50, DP1 | 256 | 24 | 0.015 | U5a1a1 | 93.6 |
| fri-1319 | Frisian | Illumina HiSeq | 17 | BQ20, MQ50, DP1 | 210 | 13 | 0.013 | H11a | 98.2 |
| fri-1320 | Frisian | Illumina HiSeq | 23 | BQ20, MQ50, DP1 | 60 | 17 | 0.004 | V3c | 98.4 |
| fri-1324 | Frisian | Illumina HiSeq | 23 | BQ20, MQ50, DP1 | 76 | 14 | 0.005 | H1a3a | 96.8 |
| fri-1325 | Frisian | Illumina HiSeq | 24 | BQ20, MQ50, DP1 | 16 | 28 | 0.001 | U5a1a1 | 96.5 |
| fri-1329 | Frisian | Illumina HiSeq | 30 | BQ20, MQ50, DP1 | 49 | 33 | 0.003 | K1a4a1b | 99.4 |
| fri-1516 | Frisian | Illumina HiSeq | 19 | BQ20, MQ50, DP1 | 11 | 35 | 0.001 | I3a | 96.7 |
| fri-1718 | Frisian | Illumina HiSeq | 22 | BQ20, MQ50, DP1 | 207 | 13 | 0.012 | H11a | 98.2 |
| fri-1722 | Frisian | Illumina HiSeq | 25 | BQ20, MQ50, DP1 | 4 | 13 | 0.000 | H31 | 98.5 |
| fri-1725 | Frisian | Illumina HiSeq | 23 | BQ20, MQ50, DP1 | 193 | 33 | 0.012 | J1c7a | 96.9 |
| fri-1784 | Frisian | IonTorrent | 78 | BQ20, MQ50, DP2 | 1 | 17 | 0.000 | H5a6 | 89.7 |
| fri-1788 | Frisian | Illumina HiSeq | 33 | BQ20, MQ50, DP1 | 10 | 8 | 0.001 | H | 98.6 |
| fri-1937 | Frisian | Illumina HiSeq | 18 | BQ20, MQ50, DP1 | 249 | 33 | 0.015 | T1a | 96.3 |
| fri-1938 | Frisian | IonTorrent | 57 | BQ20, MQ50, DP2 | 6 | 30 | 0.000 | U5a2c3a | 93.2 |
| gre-1 | Greek | IonTorrent | 103 | BQ20, MQ50, DP2 | 2 | 33 | 0.000 | T1a1l | 100 |
| gre-10 | Greek | Illumina HiSeq | 19 | BQ20, MQ50, DP1 | 111 | 12 | 0.007 | H7c4 | 91.7 |
| gre-11 | Greek | Illumina MiSeq | 2702 | BQ20, MQ50, DP2 | 0 | 34 | 0.000 | K1a12a1a | 96.8 |
| gre-12 | Greek | Illumina HiSeq | 13 | BQ20, MQ50, DP1 | 350 | 27 | 0.021 | J1c | 95.8 |
| gre-15 | Greek | IonTorrent | 73 | BQ20, MQ50, DP2 | 6 | 45 | 0.000 | M1a1 | 94.7 |
| gre-17 | Greek | Illumina HiSeq | 19 | BQ20, MQ50, DP1 | 270 | 31 | 0.016 | X1 | 91.6 |
| gre-2 | Greek | IonTorrent | 43 | BQ20, MQ50, DP2 | 53 | 13 | 0.003 | H9a | 95.7 |
| gre-3 | Greek | Illumina HiSeq | 17 | BQ20, MQ50, DP1 | 188 | 16 | 0.011 | H8a | 96.5 |
| gre-4 | Greek | Illumina HiSeq | 20 | BQ20, MQ50, DP1 | 282 | 35 | 0.017 | K1a | 90.1 |
| gre-5 | Greek | Illumina HiSeq | 21 | BQ20, MQ50, DP1 | 133 | 34 | 0.008 | T1a1l | 98.2 |
| gre-77 | Greek | Illumina MiSeq | 3503 | BQ20, MQ50, DP2 | 0 | 38 | 0.000 | N1b1a4 | 96.6 |
| gre-78 | Greek | IonTorrent | 78 | BQ20, MQ50, DP2 | 2 | 26 | 0.000 | U6a1a1 | 100 |
| gre-79 | Greek | Illumina HiSeq | 18 | BQ20, MQ50, DP1 | 330 | 10 | 0.020 | H1 | 95.7 |
| gre-80 | Greek | Illumina HiSeq | 19 | BQ20, MQ50, DP1 | 207 | 18 | 0.012 | HV1b | 86.6 |
| gre-82 | Greek | Illumina HiSeq | 15 | BQ20, MQ50, DP1 | 410 | 13 | 0.025 | V | 87.6 |
| gre-83m | Greek | Illumina MiSeq | 3159 | BQ20, MQ50, DP2 | 0 | 15 | 0.000 | V6 | 96.6 |
| gre-84m | Greek | Illumina MiSeq | 3689 | BQ20, MQ50, DP2 | 0 | 35 | 0.000 | J1c7a | 96.4 |

| | | | | | | | | | |
|---|---|---|---|---|---|---|---|---|---|
| gre-85m | Greek | Illumina MiSeq | 3164 | BQ20, MQ50, DP2 | 0 | 34 | 0.000 | T1b3 | 98.8 |
| gre-86 | Greek | Illumina MiSeq | 2204 | BQ20, MQ50, DP2 | 0 | 32 | 0.000 | T2 | 93.9 |
| gre-87 | Greek | IonTorrent | 57 | BQ20, MQ50, DP2 | 3 | 26 | 0.000 | U5a1a1 | 98.2 |
| hun-1 | Hungarian | IonTorrent | 76 | BQ20, MQ50, DP2 | 4 | 34 | 0.000 | T2b5 | 99.3 |
| hun-2 | Hungarian | Illumina MiSeq | 2305 | BQ20, MQ50, DP2 | 0 | 36 | 0.000 | U4c1a | 97.9 |
| hun-25 | Hungarian | IonTorrent | 40 | BQ20, MQ50, DP2 | 139 | 12 | 0.008 | H1c20 | 93.4 |
| hun-26 | Hungarian | Illumina MiSeq | 2647 | BQ20, MQ50, DP2 | 0 | 40 | 0.000 | J2a1a1a3 | 98.3 |
| hun-27 | Hungarian | Illumina MiSeq | 2342 | BQ20, MQ50, DP2 | 0 | 2 | 0.000 | H2a2a1 | 50 |
| hun-28 | Hungarian | Illumina MiSeq | 3914 | BQ20, MQ50, DP2 | 0 | 36 | 0.000 | K2b1b | 99.6 |
| hun-29 | Hungarian | IonTorrent | 77 | BQ20, MQ50, DP2 | 3 | 30 | 0.000 | U8b1b | 92.1 |
| hun-3 | Hungarian | Illumina MiSeq | 2971 | BQ20, MQ50, DP2 | 0 | 32 | 0.000 | U6a5 | 90 |
| hun-33 | Hungarian | Illumina MiSeq | 2148 | BQ20, MQ50, DP2 | 0 | 9 | 0.000 | H24a | 100 |
| hun-34 | Hungarian | Illumina MiSeq | 3659 | BQ20, MQ50, DP2 | 0 | 11 | 0.000 | HV | 94.6 |
| hun-35 | Hungarian | Illumina HiSeq | 29 | BQ20, MQ50, DP1 | 482 | 31 | 0.029 | T2b | 100 |
| hun-37 | Hungarian | Illumina HiSeq | 14 | BQ20, MQ50, DP1 | 190 | 32 | 0.011 | I4a1 | 95.4 |
| hun-38 | Hungarian | Illumina MiSeq | 3419 | BQ20, MQ50, DP2 | 0 | 10 | 0.000 | H1 | 94.3 |
| hun-39 | Hungarian | Illumina HiSeq | 24 | BQ20, MQ50, DP1 | 221 | 15 | 0.013 | H11a2a | 90.1 |
| hun-40 | Hungarian | IonTorrent | 81 | BQ20, MQ50, DP2 | 1 | 34 | 0.000 | T1b4 | 91.9 |
| hun-43 | Hungarian | Illumina MiSeq | 4530 | BQ20, MQ50, DP2 | 0 | 10 | 0.000 | H20 | 89.9 |
| hun-46 | Hungarian | Illumina HiSeq | 22 | BQ20, MQ50, DP1 | 205 | 11 | 0.012 | H1 | 89.6 |
| hun-47 | Hungarian | Illumina MiSeq | 1983 | BQ20, MQ50, DP2 | 0 | 11 | 0.000 | H1 | 92.7 |
| hun-5 | Hungarian | IonTorrent | 57 | BQ20, MQ50, DP2 | 2 | 32 | 0.000 | K2a10 | 98.7 |
| hun-9 | Hungarian | Illumina MiSeq | 3464 | BQ20, MQ50, DP2 | 0 | 8 | 0.000 | H10b | 100 |
| ire-0052 | Irish | Illumina MiSeq | 2035 | BQ20, MQ50, DP2 | 0 | 16 | 0.000 | H6a1b4 | 95.7 |
| ire-0053 | Irish | IonTorrent | 39 | BQ20, MQ50, DP2 | 106 | 17 | 0.006 | V1a | 96.8 |
| ire-0059 | Irish | Illumina HiSeq | 23 | BQ20, MQ50, DP1 | 305 | 37 | 0.018 | T2b3b | 98.1 |
| ire-0068 | Irish | IonTorrent | 67 | BQ20, MQ50, DP2 | 7 | 32 | 0.000 | J1c3e1 | 97.5 |
| ire-0078 | Irish | Illumina MiSeq | 3040 | BQ20, MQ50, DP2 | 0 | 30 | 0.000 | J1c1b2 | 99.1 |
| ire-0093 | Irish | Illumina MiSeq | 2406 | BQ20, MQ50, DP2 | 0 | 38 | 0.000 | I1a1 | 98.1 |
| ire-0095 | Irish | IonTorrent | 63 | BQ20, MQ50, DP2 | 1 | 11 | 0.000 | H56a1 | 100 |
| ire-0100 | Irish | Illumina MiSeq | 3774 | BQ20, MQ50, DP2 | 0 | 9 | 0.000 | H3 | 93.4 |
| ire-0107 | Irish | Illumina HiSeq | 20 | BQ20, MQ50, DP1 | 442 | 11 | 0.027 | H16 | 97.9 |
| ire-0109 | Irish | Illumina HiSeq | 23 | BQ20, MQ50, DP1 | 109 | 31 | 0.007 | K2a10 | 100 |
| ire-0114 | Irish | IonTorrent | 73 | BQ20, MQ50, DP2 | 0 | 14 | 0.000 | H1m1 | 97 |
| ire-0116 | Irish | IonTorrent | 84 | BQ20, MQ50, DP2 | 1 | 11 | 0.000 | H1c1 | 100 |
| ire-0120 | Irish | Illumina HiSeq | 19 | BQ20, MQ50, DP1 | 438 | 30 | 0.026 | K1a12 | 97.7 |
| ire-0130 | Irish | Illumina MiSeq | 3567 | BQ20, MQ50, DP2 | 0 | 13 | 0.000 | H3a1 | 96.2 |
| ire-0136 | Irish | Illumina MiSeq | 3455 | BQ20, MQ50, DP2 | 0 | 31 | 0.000 | K2a6 | 100 |
| ire-112 | Irish | Illumina HiSeq | 14 | BQ20, MQ50, DP1 | 608 | 37 | 0.037 | K1c1 | 92.8 |
| ire-132 | Irish | Illumina HiSeq | 17 | BQ20, MQ50, DP1 | 441 | 40 | 0.027 | U2e3a | 95 |
| ire-55 | Irish | Illumina MiSeq | 1821 | BQ20, MQ50, DP2 | 0 | 33 | 0.000 | T1a1 | 100 |
| ire-74 | Irish | Illumina MiSeq | 1622 | BQ20, MQ50, DP2 | 0 | 28 | 0.000 | X2b+226 | 100 |
| ire-94 | Irish | Illumina HiSeq | 22 | BQ20, MQ50, DP1 | 209 | 40 | 0.013 | U2e3a | 94.5 |
| TSI-NA20510 | Italian (TSI) | Illumina HiSeq | 87 | BQ20, MQ50, DP1 | 1 | 33 | 0.000 | W6 | 96.6 |
| TSI-NA20538 | Italian (TSI) | Illumina HiSeq | 100 | BQ20, MQ50, DP1 | 4 | 10 | 0.000 | H82 | 89.8 |
| TSI-NA20543 | Italian (TSI) | Illumina HiSeq | 72 | BQ20, MQ50, DP1 | 2 | 16 | 0.000 | H1au | 81.5 |
| TSI-NA20581 | Italian (TSI) | Illumina HiSeq | 79 | BQ20, MQ50, DP1 | 6 | 38 | 0.000 | T1a2a | 100 |
| TSI-NA20586 | Italian (TSI) | Illumina HiSeq | 65 | BQ20, MQ50, DP1 | 2 | 11 | 0.000 | H1ba | 92.2 |
| TSI-NA20588 | Italian (TSI) | Illumina HiSeq | 78 | BQ20, MQ50, DP1 | 3 | 11 | 0.000 | H23 | 91.8 |
| TSI-NA20752 | Italian (TSI) | Illumina HiSeq | 55 | BQ20, MQ50, DP1 | 6 | 35 | 0.000 | J2a2b2 | 100 |
| TSI-NA20758 | Italian (TSI) | Illumina HiSeq | 141 | BQ20, MQ50, DP1 | 0 | 39 | 0.000 | T2c1d | 97.1 |
| TSI-NA20759 | Italian (TSI) | Illumina HiSeq | 65 | BQ20, MQ50, DP1 | 1 | 40 | 0.000 | K1a1b2a1a | 97.1 |
| TSI-NA20765 | Italian (TSI) | Illumina HiSeq | 91 | BQ20, MQ50, DP1 | 1 | 28 | 0.000 | U5a2b2 | 99.8 |
| TSI-NA20783 | Italian (TSI) | Illumina HiSeq | 146 | BQ20, MQ50, DP1 | 3 | 36 | 0.000 | T2c1a2 | 95.5 |
| TSI-NA20785 | Italian (TSI) | Illumina HiSeq | 124 | BQ20, MQ50, DP1 | 1 | 31 | 0.000 | J1c3e2 | 98.1 |
| TSI-NA20792 | Italian (TSI) | Illumina HiSeq | 83 | BQ20, MQ50, DP1 | 1 | 13 | 0.000 | H1b1 | 95.3 |
| TSI-NA20796 | Italian (TSI) | Illumina HiSeq | 59 | BQ20, MQ50, DP1 | 7 | 12 | 0.000 | H7 | 85.4 |
| TSI-NA20798 | Italian (TSI) | Illumina HiSeq | 71 | BQ20, MQ50, DP1 | 3 | 12 | 0.000 | H15b1 | 100 |
| TSI-NA20803 | Italian (TSI) | Illumina HiSeq | 60 | BQ20, MQ50, DP1 | 5 | 13 | 0.000 | H1e2d | 93.5 |
| TSI-NA20805 | Italian (TSI) | Illumina HiSeq | 43 | BQ20, MQ50, DP1 | 2 | 27 | 0.000 | U5a1g | 92.9 |
| TSI-NA20806 | Italian (TSI) | Illumina HiSeq | 54 | BQ20, MQ50, DP1 | 3 | 18 | 0.000 | H13b1b | 93.5 |
| TSI-NA20810 | Italian (TSI) | Illumina HiSeq | 64 | BQ20, MQ50, DP1 | 0 | 17 | 0.000 | H1e | 81.7 |
| TSI-NA20815 | Italian (TSI) | Illumina HiSeq | 49 | BQ20, MQ50, DP1 | 1 | 10 | 0.000 | H44a | 100 |
| nor-13 | Norwegian | IonTorrent | 104 | BQ20, MQ50, DP2 | 0 | 14 | 0.000 | V | 100 |
| nor-14 | Norwegian | Illumina HiSeq | 27 | BQ20, MQ50, DP1 | 160 | 37 | 0.010 | T2a1a | 96.4 |
| nor-15 | Norwegian | Illumina HiSeq | 35 | BQ20, MQ50, DP1 | 84 | 34 | 0.005 | K1a3 | 96.1 |
| nor-16 | Norwegian | IonTorrent | 36 | BQ20, MQ50, DP2 | 27 | 9 | 0.002 | H2a1 | 87.1 |
| nor-17 | Norwegian | Illumina MiSeq | 3239 | BQ20, MQ50, DP2 | 0 | 27 | 0.000 | U5a1a1 | 99.3 |
| nor-18 | Norwegian | Illumina MiSeq | 2712 | BQ20, MQ50, DP2 | 0 | 37 | 0.000 | D2 | 95.4 |
| nor-2 | Norwegian | Illumina MiSeq | 2216 | BQ20, MQ50, DP2 | 0 | 14 | 0.000 | H13a1a1a | 99.6 |
| nor-20 | Norwegian | IonTorrent | 71 | BQ20, MQ50, DP2 | 1 | 13 | 0.000 | H6a1a | 100 |

| | | | | | | | | | |
|---|---|---|---|---|---|---|---|---|---|
| nor-21 | Norwegian | Illumina MiSeq | 3215 | BQ20, MQ50, DP2 | 0 | 32 | 0.000 | K1c1b | 98.4 |
| nor-23 | Norwegian | Illumina HiSeq | 28 | BQ20, MQ50, DP1 | 152 | 38 | 0.009 | K2a11 | 91.6 |
| nor-24 | Norwegian | Illumina HiSeq | 36 | BQ20, MQ50, DP1 | 77 | 33 | 0.005 | T2b2b | 95.9 |
| nor-25 | Norwegian | Illumina MiSeq | 1654 | BQ20, MQ50, DP2 | 0 | 28 | 0.000 | U5a1b1e | 97.4 |
| nor-26 | Norwegian | Illumina MiSeq | 2890 | BQ20, MQ50, DP2 | 0 | 27 | 0.000 | U5b1b2 | 97.1 |
| nor-27 | Norwegian | Illumina MiSeq | 1508 | BQ20, MQ50, DP2 | 0 | 6 | 0.000 | H2a5 | 100 |
| nor-28 | Norwegian | Illumina HiSeq | 21 | BQ20, MQ50, DP1 | 126 | 33 | 0.008 | K1c2 | 98.4 |
| nor-29 | Norwegian | IonTorrent | 65 | BQ20, MQ50, DP2 | 1 | 28 | 0.000 | U5a1b1e | 97.4 |
| nor-3 | Norwegian | Illumina HiSeq | 34 | BQ20, MQ50, DP1 | 45 | 38 | 0.003 | T2a1b1a1 | 98 |
| nor-7 | Norwegian | Illumina MiSeq | 3213 | BQ20, MQ50, DP2 | 0 | 33 | 0.000 | T2b2b | 95.9 |
| nor-8 | Norwegian | Illumina MiSeq | 2206 | BQ20, MQ50, DP2 | 0 | 13 | 0.000 | V3c | 96.5 |
| nor-9 | Norwegian | IonTorrent | 62 | BQ20, MQ50, DP2 | 1 | 39 | 0.000 | T2b25 | 95.7 |
| ork-004 | Orcadian | IonTorrent | 68 | BQ20, MQ50, DP2 | 7 | 28 | 0.000 | U5b2b2 | 100 |
| ork-007 | Orcadian | IonTorrent | 65 | BQ20, MQ50, DP2 | 2 | 11 | 0.000 | H5b2 | 100 |
| ork-008 | Orcadian | Illumina HiSeq | 103 | BQ20, MQ50, DP1 | 1 | 37 | 0.000 | J1c2l | 95.7 |
| ork-010 | Orcadian | Illumina HiSeq | 119 | BQ20, MQ50, DP1 | 1 | 31 | 0.000 | X2b8 | 98.1 |
| ork-022m | Orcadian | Illumina HiSeq | 72 | BQ20, MQ50, DP1 | 2 | 8 | 0.000 | H23 | 100 |
| ork-026m | Orcadian | Illumina HiSeq | 77 | BQ20, MQ50, DP1 | 2 | 10 | 0.000 | H66a | 93.4 |
| ork-036m | Orcadian | Illumina HiSeq | 94 | BQ20, MQ50, DP1 | 1 | 34 | 0.000 | I2 | 96.7 |
| ork-525 | Orcadian | IonTorrent | 58 | BQ20, MQ50, DP2 | 4 | 31 | 0.000 | J1c2e1 | 96.1 |
| ork-552 | Orcadian | Illumina HiSeq | 109 | BQ20, MQ50, DP1 | 6 | 33 | 0.000 | T1a1 | 95.9 |
| ork-564 | Orcadian | Illumina HiSeq | 157 | BQ20, MQ50, DP1 | 0 | 29 | 0.000 | X2b | 96.8 |
| ork-565 | Orcadian | Illumina HiSeq | 66 | BQ20, MQ50, DP1 | 2 | 28 | 0.000 | U4a2 | 98.5 |
| ork-567 | Orcadian | Illumina HiSeq | 91 | BQ20, MQ50, DP1 | 2 | 39 | 0.000 | J1b1a1a | 98.1 |
| ork-571 | Orcadian | Illumina HiSeq | 53 | BQ20, MQ50, DP1 | 24 | 12 | 0.001 | H16a1 | 97 |
| ork-573 | Orcadian | IonTorrent | 47 | BQ20, MQ50, DP2 | 5 | 29 | 0.000 | J1c3g | 96.8 |
| ork-575 | Orcadian | Illumina HiSeq | 131 | BQ20, MQ50, DP1 | 1 | 11 | 0.000 | H5b2 | 100 |
| ork-577 | Orcadian | Illumina HiSeq | 59 | BQ20, MQ50, DP1 | 5 | 37 | 0.000 | T2b23a | 98.7 |
| ork-579 | Orcadian | Illumina HiSeq | 64 | BQ20, MQ50, DP1 | 4 | 31 | 0.000 | U5b2c2b | 98.2 |
| ork-583 | Orcadian | Illumina HiSeq | 58 | BQ20, MQ50, DP1 | 28 | 31 | 0.002 | X2b8 | 98.1 |
| ork-584 | Orcadian | Illumina HiSeq | 70 | BQ20, MQ50, DP1 | 1 | 31 | 0.000 | U5b2c2b | 98.2 |
| ork-585 | Orcadian | IonTorrent | 53 | BQ20, MQ50, DP2 | 1 | 30 | 0.000 | J1c3b1a | 98.8 |
| pal-4918 | Palestinian | Illumina HiSeq | 65 | BQ20, MQ50, DP1 | 3 | 37 | 0.000 | T2b | 90.3 |
| pal-4919 | Palestinian | Illumina HiSeq | 134 | BQ20, MQ50, DP1 | 1 | 30 | 0.000 | U6a3d1a | 98.5 |
| pal-4921 | Palestinian | Illumina HiSeq | 75 | BQ20, MQ50, DP1 | 3 | 34 | 0.000 | L3e3a | 97.8 |
| pal-4922 | Palestinian | Illumina HiSeq | 82 | BQ20, MQ50, DP1 | 2 | 28 | 0.000 | M30 | 96.6 |
| pal-4929 | Palestinian | Illumina HiSeq | 107 | BQ20, MQ50, DP1 | 3 | 15 | 0.000 | HV1a'b'c | 95 |
| pal-4940 | Palestinian | Illumina HiSeq | 102 | BQ20, MQ50, DP1 | 0 | 35 | 0.000 | I | 89.8 |
| pal-4941 | Palestinian | Illumina HiSeq | 101 | BQ20, MQ50, DP1 | 4 | 37 | 0.000 | U1a1b | 95 |
| pal-4942 | Palestinian | Illumina HiSeq | 123 | BQ20, MQ50, DP1 | 1 | 54 | 0.000 | L2a1 | 95 |
| pal-4943 | Palestinian | Illumina HiSeq | 119 | BQ20, MQ50, DP1 | 2 | 9 | 0.000 | H | 92.6 |
| pal-4947 | Palestinian | Illumina HiSeq | 155 | BQ20, MQ50, DP1 | 3 | 18 | 0.000 | HV1 | 83.3 |
| pal-5047 | Palestinian | IonTorrent | 89 | BQ20, MQ50, DP2 | 2 | 27 | 0.000 | U5a1a1 | 98.2 |
| pal-5048 | Palestinian | Illumina HiSeq | 91 | BQ20, MQ50, DP1 | 1 | 14 | 0.000 | HV0c | 92.6 |
| pal-5225 | Palestinian | Illumina HiSeq | 109 | BQ20, MQ50, DP1 | 3 | 35 | 0.000 | J1b1a1 | 99.6 |
| pal-5232 | Palestinian | Illumina HiSeq | 96 | BQ20, MQ50, DP1 | 1 | 39 | 0.000 | T2a1b1a1b | 93.3 |
| pal-5257 | Palestinian | Illumina HiSeq | 97 | BQ20, MQ50, DP1 | 1 | 38 | 0.000 | K1b1c | 97.6 |
| pal-5341 | Palestinian | Illumina HiSeq | 97 | BQ20, MQ50, DP1 | 3 | 10 | 0.000 | H1 | 93.2 |
| pal-5365 | Palestinian | Illumina HiSeq | 167 | BQ20, MQ50, DP1 | 1 | 32 | 0.000 | L3e1 | 94.8 |
| pal-5366 | Palestinian | Illumina HiSeq | 102 | BQ20, MQ50, DP1 | 1 | 27 | 0.000 | U5a2a1 | 98.1 |
| pal-5370 | Palestinian | IonTorrent | 70 | BQ20, MQ50, DP2 | 2 | 36 | 0.000 | U1a1 | 96.2 |
| pal-5420 | Palestinian | Illumina HiSeq | 73 | BQ20, MQ50, DP1 | 7 | 41 | 0.000 | N1b1a | 95.4 |
| saa-1 | Saami | Illumina MiSeq | 2381 | BQ20, MQ50, DP2 | 0 | 30 | 0.000 | U5b1b1a | 96.4 |
| saa-10 | Saami | IonTorrent | 80 | BQ20, MQ50, DP2 | 2 | 26 | 0.000 | U5b1b1a | 97.7 |
| saa-11 | Saami | IonTorrent | 43 | BQ20, MQ50, DP2 | 4 | 17 | 0.000 | V7a1 | 100 |
| saa-12 | Saami | IonTorrent | 101 | BQ20, MQ50, DP2 | 12 | 40 | 0.001 | D5a3a1a | 99.4 |
| saa-13 | Saami | Illumina HiSeq | 25 | BQ20, MQ50, DP1 | 209 | 30 | 0.013 | U5b1b1a | 95.4 |
| saa-14 | Saami | Illumina MiSeq | 2860 | BQ20, MQ50, DP2 | 0 | 27 | 0.000 | U5b1b1a | 100 |
| saa-15 | Saami | Illumina MiSeq | 2010 | BQ20, MQ50, DP2 | 0 | 12 | 0.000 | V | 100 |
| saa-16 | Saami | Illumina MiSeq | 2604 | BQ20, MQ50, DP2 | 0 | 30 | 0.000 | U5b1b1a | 95.4 |
| saa-17 | Saami | Illumina MiSeq | 2544 | BQ20, MQ50, DP2 | 0 | 12 | 0.000 | V | 100 |
| saa-18 | Saami | Illumina HiSeq | 21 | BQ20, MQ50, DP1 | 532 | 29 | 0.032 | U5b1b1a | 92.8 |
| saa-19 | Saami | Illumina MiSeq | 1972 | BQ20, MQ50, DP2 | 1 | 29 | 0.000 | U5b1b1a | 96.8 |
| saa-2 | Saami | Illumina HiSeq | 19 | BQ20, MQ50, DP1 | 348 | 28 | 0.021 | U5b1b1a | 91 |
| saa-20 | Saami | IonTorrent | 73 | BQ20, MQ50, DP2 | 1 | 31 | 0.000 | U5b1b1a | 94.5 |
| saa-3 | Saami | IonTorrent | 77 | BQ20, MQ50, DP2 | 14 | 17 | 0.001 | V7a1 | 100 |
| saa-4 | Saami | Illumina MiSeq | 3111 | BQ20, MQ50, DP2 | 0 | 30 | 0.000 | U5b1b1a | 95.4 |
| saa-5 | Saami | Illumina HiSeq | 33 | BQ20, MQ50, DP1 | 106 | 28 | 0.006 | U5b1b1a | 97.6 |
| saa-6 | Saami | Illumina MiSeq | 1927 | BQ20, MQ50, DP2 | 0 | 29 | 0.000 | U5b1b1a | 98 |
| saa-7 | Saami | Illumina MiSeq | 2687 | BQ20, MQ50, DP2 | 0 | 29 | 0.000 | U5b1b1a | 96.8 |
| saa-8 | Saami | Illumina MiSeq | 2048 | BQ20, MQ50, DP2 | 0 | 30 | 0.000 | U5b1b1a | 96.8 |

| | | | | | | | | | |
|---|---|---|---|---|---|---|---|---|---|
| saa-9 | Saami | Illumina MiSeq | 2521 | BQ20, MQ50, DP2 | 0 | 17 | 0.000 | V7a1 | 100 |
| ser-1 | Serbian | IonTorrent | 70 | BQ20, MQ50, DP2 | 10 | 28 | 0.001 | U4a | 98 |
| ser-12 | Serbian | IonTorrent | 87 | BQ20, MQ50, DP2 | 2 | 36 | 0.000 | T2a1a1 | 96 |
| ser-13 | Serbian | IonTorrent | 57 | BQ20, MQ50, DP2 | 3 | 34 | 0.000 | U4b1a1a1 | 98.5 |
| ser-16 | Serbian | Illumina MiSeq | 1734 | BQ20, MQ50, DP2 | 0 | 15 | 0.000 | H6a1a | 100 |
| ser-19 | Serbian | Illumina HiSeq | 12 | BQ20, MQ50, DP1 | 310 | 11 | 0.019 | H12 | 96.9 |
| ser-2 | Serbian | Illumina HiSeq | 11 | BQ20, MQ50, DP1 | 178 | 36 | 0.011 | I1a1 | 95 |
| ser-20 | Serbian | Illumina HiSeq | 24 | BQ20, MQ50, DP1 | 54 | 37 | 0.003 | T2b | 93.8 |
| ser-21 | Serbian | IonTorrent | 54 | BQ20, MQ50, DP2 | 8 | 11 | 0.000 | H83 | 88.2 |
| ser-23 | Serbian | Illumina HiSeq | 24 | BQ20, MQ50, DP1 | 148 | 28 | 0.009 | U4a | 93 |
| ser-24 | Serbian | Illumina HiSeq | 21 | BQ20, MQ50, DP1 | 837 | 9 | 0.051 | H | 91.4 |
| ser-26 | Serbian | Illumina HiSeq | 15 | BQ20, MQ50, DP1 | 559 | 35 | 0.034 | T1a1l | 97.6 |
| ser-27 | Serbian | Illumina MiSeq | 2591 | BQ20, MQ50, DP2 | 0 | 12 | 0.000 | H5n | 93.1 |
| ser-28 | Serbian | Illumina MiSeq | 1846 | BQ20, MQ50, DP2 | 0 | 33 | 0.000 | U2d | 94.2 |
| ser-3 | Serbian | Illumina MiSeq | 2097 | BQ20, MQ50, DP2 | 0 | 10 | 0.000 | H7g | 94.8 |
| ser-30 | Serbian | IonTorrent | 74 | BQ20, MQ50, DP2 | 5 | 30 | 0.000 | J2b1c | 100 |
| ser-4 | Serbian | Illumina MiSeq | 1850 | BQ20, MQ50, DP2 | 0 | 33 | 0.000 | T2b2b1 | 98.3 |
| ser-5 | Serbian | Illumina MiSeq | 1709 | BQ20, MQ50, DP2 | 0 | 14 | 0.000 | H1u | 86.4 |
| ser-6 | Serbian | Illumina MiSeq | 2120 | BQ20, MQ50, DP2 | 0 | 33 | 0.000 | T2b4a | 98.2 |
| ser-7 | Serbian | Illumina MiSeq | 2152 | BQ20, MQ50, DP2 | 0 | 29 | 0.000 | U4a2a | 95 |
| ser-9 | Serbian | Illumina MiSeq | 2771 | BQ20, MQ50, DP2 | 0 | 13 | 0.000 | H13a1a1e | 100 |
| spa-14 | Spanish | IonTorrent | 151 | BQ20, MQ50, DP2 | 1 | 30 | 0.000 | J1b2 | 96.7 |
| spa-15 | Spanish | Illumina MiSeq | 2025 | BQ20, MQ50, DP2 | 0 | 10 | 0.000 | H1 | 95.9 |
| spa-2 | Spanish | Illumina MiSeq | 1916 | BQ20, MQ50, DP2 | 0 | 29 | 0.000 | U5a1i | 98.1 |
| spa-20 | Spanish | Illumina MiSeq | 1831 | BQ20, MQ50, DP2 | 0 | 13 | 0.000 | H1c | 92.1 |
| spa-22 | Spanish | Illumina MiSeq | 2067 | BQ20, MQ50, DP2 | 0 | 39 | 0.000 | K1a | 87.7 |
| spa-25 | Spanish | Illumina MiSeq | 1681 | BQ20, MQ50, DP2 | 0 | 11 | 0.000 | H3 | 93.5 |
| spa-26u | Spanish | Illumina HiSeq | 19 | BQ20, MQ50, DP1 | 291 | 13 | 0.018 | H1c4a | 95.3 |
| spa-31 | Spanish | IonTorrent | 86 | BQ20, MQ50, DP2 | 1 | 31 | 0.000 | K2a | 100 |
| spa-32 | Spanish | Illumina MiSeq | 1065 | BQ20, MQ50, DP2 | 0 | 33 | 0.000 | J1c3 | 97.8 |
| spa-37 | Spanish | Illumina MiSeq | 2150 | BQ20, MQ50, DP2 | 0 | 57 | 0.000 | L2a1 | 94.2 |
| spa-40 | Spanish | Illumina MiSeq | 3299 | BQ20, MQ50, DP2 | 0 | 11 | 0.000 | H1e1a | 100 |
| spa-41 | Spanish | Illumina HiSeq | 19 | BQ20, MQ50, DP1 | 302 | 31 | 0.018 | U5a1a1 | 90.8 |
| spa-47 | Spanish | IonTorrent | 58 | BQ20, MQ50, DP2 | 1 | 15 | 0.000 | HV0f | 88.7 |
| spa-51 | Spanish | Illumina MiSeq | 2385 | BQ20, MQ50, DP2 | 0 | 16 | 0.000 | H13a2c | 92.4 |
| spa-53 | Spanish | Illumina MiSeq | 2000 | BQ20, MQ50, DP2 | 0 | 15 | 0.000 | H8c | 99 |
| spa-54u | Spanish | IonTorrent | 33 | BQ20, MQ50, DP2 | 111 | 12 | 0.007 | V | 100 |
| spa-60 | Spanish | Illumina MiSeq | 1888 | BQ20, MQ50, DP2 | 0 | 32 | 0.000 | J1c2e2 | 98.8 |
| spa-60u | Spanish | IonTorrent | 68 | BQ20, MQ50, DP2 | 6 | 34 | 0.000 | K1a1b1 | 93.2 |
| spa-62 | Spanish | Illumina MiSeq | 2009 | BQ20, MQ50, DP2 | 0 | 7 | 0.000 | H3 | 100 |
| spa-63 | Spanish | Illumina MiSeq | 2201 | BQ20, MQ50, DP2 | 0 | 11 | 0.000 | H1c4 | 95 |
| tur-1 | Turkish | IonTorrent | 35 | BQ20, MQ50, DP2 | 420 | 24 | 0.025 | X2f | 98.4 |
| tur-10 | Turkish | IonTorrent | 87 | BQ20, MQ50, DP2 | 13 | 29 | 0.001 | A | 90.6 |
| tur-11 | Turkish | IonTorrent | 62 | BQ20, MQ50, DP2 | 7 | 15 | 0.000 | H13a2b2 | 87.8 |
| tur-12 | Turkish | Illumina HiSeq | 15 | BQ20, MQ50, DP1 | 255 | 34 | 0.015 | U2d2 | 91.4 |
| tur-13 | Turkish | Illumina HiSeq | 20 | BQ20, MQ50, DP1 | 343 | 34 | 0.021 | T2a1b | 94.2 |
| tur-14 | Turkish | Illumina MiSeq | 2318 | BQ20, MQ50, DP2 | 0 | 41 | 0.000 | I1c1a | 96.3 |
| tur-15 | Turkish | Illumina MiSeq | 1763 | BQ20, MQ50, DP2 | 0 | 34 | 0.000 | T2b13a | 100 |
| tur-16 | Turkish | IonTorrent | 65 | BQ20, MQ50, DP2 | 2 | 24 | 0.000 | HV2a | 84.9 |
| tur-17 | Turkish | Illumina MiSeq | 2076 | BQ20, MQ50, DP2 | 0 | 29 | 0.000 | U4a2b | 98.1 |
| tur-18 | Turkish | Illumina HiSeq | 22 | BQ20, MQ50, DP1 | 198 | 26 | 0.012 | U5a1a1 | 94 |
| tur-19 | Turkish | Illumina MiSeq | 3092 | BQ20, MQ50, DP2 | 0 | 34 | 0.000 | J1b3b | 96.3 |
| tur-2 | Turkish | Illumina MiSeq | 1891 | BQ20, MQ50, DP2 | 0 | 9 | 0.000 | H | 87.8 |
| tur-21 | Turkish | IonTorrent | 66 | BQ20, MQ50, DP2 | 6 | 35 | 0.000 | N1a3 | 91.6 |
| tur-3 | Turkish | Illumina MiSeq | 1590 | BQ20, MQ50, DP2 | 3 | 18 | 0.000 | HV4 | 83.6 |
| tur-4 | Turkish | Illumina MiSeq | 2486 | BQ20, MQ50, DP2 | 0 | 37 | 0.000 | T2b | 93.5 |
| tur-5 | Turkish | Illumina MiSeq | 2572 | BQ20, MQ50, DP2 | 1 | 46 | 0.000 | C4a1d | 95.4 |
| tur-6 | Turkish | Illumina MiSeq | 1703 | BQ20, MQ50, DP2 | 0 | 10 | 0.000 | H5 | 94.3 |
| tur-7 | Turkish | Illumina MiSeq | 2727 | BQ20, MQ50, DP2 | 0 | 33 | 0.000 | J1d3b | 96 |
| tur-8 | Turkish | Illumina MiSeq | 2258 | BQ20, MQ50, DP2 | 0 | 34 | 0.000 | U1b | 94.1 |
| tur-9 | Turkish | Illumina MiSeq | 2285 | BQ20, MQ50, DP2 | 0 | 25 | 0.000 | R0a2 | 88.8 |

| | | | | | | | | | |
|---|---|---|---|---|---|---|---|---|---|
| CHB-NA18558 | CHB | Illumina HiSeq | 64 | BQ20, MQ50, DP1 | 3 | 38 | 0.000 | D5a2a2 | 98.3 |
| CHB-NA18561 | CHB | Illumina HiSeq | 88 | BQ20, MQ50, DP1 | 5 | 29 | 0.000 | F1d | 90 |
| CHB-NA18562 | CHB | Illumina HiSeq | 76 | BQ20, MQ50, DP1 | 4 | 31 | 0.000 | D4i | 98.3 |
| CHB-NA18572 | CHB | Illumina HiSeq | 65 | BQ20, MQ50, DP1 | 4 | 28 | 0.000 | A17 | 99.1 |
| CHB-NA18603 | CHB | Illumina HiSeq | 74 | BQ20, MQ50, DP1 | 4 | 30 | 0.000 | F1d1 | 96.5 |
| CHB-NA18605 | CHB | Illumina HiSeq | 106 | BQ20, MQ50, DP1 | 1 | 44 | 0.000 | D5c1 | 94.7 |
| CHB-NA18608 | CHB | Illumina HiSeq | 97 | BQ20, MQ50, DP1 | 1 | 40 | 0.000 | M11c | 92.7 |
| CHB-NA18609 | CHB | Illumina HiSeq | 54 | BQ20, MQ50, DP1 | 4 | 37 | 0.000 | F2c2 | 95.4 |
| CHB-NA18611 | CHB | Illumina HiSeq | 66 | BQ20, MQ50, DP1 | 6 | 31 | 0.000 | A5b1 | 92.8 |

| | | | | | | | | | |
|---|---|---|---|---|---|---|---|---|---|
| CHB-NA18612 | CHB | Illumina HiSeq | 46 | BQ20, MQ50, DP1 | 24 | 28 | 0.001 | N9a9 | 90 |
| CHB-NA18620 | CHB | Illumina HiSeq | 49 | BQ20, MQ50, DP1 | 5 | 33 | 0.000 | F1a1c | 93.3 |
| CHB-NA18621 | CHB | Illumina HiSeq | 59 | BQ20, MQ50, DP1 | 7 | 30 | 0.000 | A19 | 95.2 |
| CHB-NA18622 | CHB | Illumina HiSeq | 64 | BQ20, MQ50, DP1 | 5 | 39 | 0.000 | Z3 | 96.9 |
| CHB-NA18623 | CHB | Illumina HiSeq | 59 | BQ20, MQ50, DP1 | 3 | 40 | 0.000 | D5a2a1+@ 16172 | 96 |
| CHB-NA18624 | CHB | Illumina HiSeq | 63 | BQ20, MQ50, DP1 | 3 | 28 | 0.000 | F2b1 | 93.7 |
| CHB-NA18632 | CHB | Illumina HiSeq | 66 | BQ20, MQ50, DP1 | 4 | 28 | 0.000 | A15 | 92.9 |
| CHB-NA18633 | CHB | Illumina HiSeq | 65 | BQ20, MQ50, DP1 | 3 | 33 | 0.000 | D4k | 96.4 |
| CHB-NA18635 | CHB | Illumina HiSeq | 79 | BQ20, MQ50, DP1 | 1 | 40 | 0.000 | G2a1f | 97.2 |
| CHB-NA18636 | CHB | Illumina HiSeq | 80 | BQ20, MQ50, DP1 | 3 | 41 | 0.000 | M7b1a1a | 97 |
| CHB-NA18637 | CHB | Illumina HiSeq | 92 | BQ20, MQ50, DP1 | 7 | 36 | 0.000 | G2b1a2 | 97.4 |
| YRI-NA18501 | YRI | Illumina HiSeq | 79 | BQ20, MQ50, DP1 | 22 | 76 | 0.001 | L1b1a18 | 98.6 |
| YRI-NA18504 | YRI | Illumina HiSeq | 109 | BQ20, MQ50, DP1 | 0 | 33 | 0.000 | L3e2b | 95.2 |
| YRI-NA18507 | YRI | Illumina HiSeq | 81 | BQ20, MQ50, DP1 | 22 | 77 | 0.001 | L1b1a3 | 100 |
| YRI-NA18516 | YRI | Illumina HiSeq | 88 | BQ20, MQ50, DP1 | 0 | 69 | 0.000 | L2b1a3 | 99.4 |
| YRI-NA18522 | YRI | Illumina HiSeq | 97 | BQ20, MQ50, DP1 | 3 | 72 | 0.000 | L2b1a3 | 98 |
| YRI-NA18853 | YRI | Illumina HiSeq | 101 | BQ20, MQ50, DP1 | 5 | 32 | 0.000 | L3e2b | 95.7 |
| YRI-NA18856 | YRI | Illumina HiSeq | 62 | BQ20, MQ50, DP1 | 5 | 56 | 0.000 | L2a1b3 | 97.4 |
| YRI-NA18871 | YRI | Illumina HiSeq | 73 | BQ20, MQ50, DP1 | 22 | 39 | 0.001 | L3b1a1 | 92.9 |
| YRI-NA19098 | YRI | Illumina HiSeq | 116 | BQ20, MQ50, DP1 | 1 | 36 | 0.000 | L3b1a | 91.6 |
| YRI-NA19119 | YRI | Illumina HiSeq | 60 | BQ20, MQ50, DP1 | 4 | 40 | 0.000 | L3b1a7a | 94.4 |
| YRI-NA19130 | YRI | Illumina HiSeq | 60 | BQ20, MQ50, DP1 | 2 | 58 | 0.000 | L2a1c3b2 | 96 |
| YRI-NA19138 | YRI | Illumina HiSeq | 67 | BQ20, MQ50, DP1 | 10 | 33 | 0.001 | L3e2b3 | 94.7 |
| YRI-NA19144 | YRI | Illumina HiSeq | 63 | BQ20, MQ50, DP1 | 4 | 37 | 0.000 | L3d5a | 96.4 |
| YRI-NA19153 | YRI | Illumina HiSeq | 96 | BQ20, MQ50, DP1 | 1 | 38 | 0.000 | L3d2b | 92.1 |
| YRI-NA19160 | YRI | Illumina HiSeq | 65 | BQ20, MQ50, DP1 | 4 | 39 | 0.000 | L3e2b2 | 92.3 |
| YRI-NA19171 | YRI | Illumina HiSeq | 65 | BQ20, MQ50, DP1 | 8 | 39 | 0.000 | L3e3b | 98.5 |
| YRI-NA19200 | YRI | Illumina HiSeq | 81 | BQ20, MQ50, DP1 | 1 | 36 | 0.000 | L3e3b | 100 |
| YRI-NA19203 | YRI | Illumina HiSeq | 102 | BQ20, MQ50, DP1 | 1 | 77 | 0.000 | L1b1a7 | 100 |
| YRI-NA19207 | YRI | Illumina HiSeq | 56 | BQ20, MQ50, DP1 | 16 | 75 | 0.001 | L1b1a3 | 99.3 |
| YRI-NA19223 | YRI | Illumina HiSeq | 61 | BQ20, MQ50, DP1 | 10 | 76 | 0.001 | L1b1a3 | 100 |

BQ: base quality; MQ: mapping quality; DP: depth; rCRS: revised Cambridge reference sequence.
YRI: Yoruba in Ibadan, Nigeria; CHB: Han Chinese in Beijing, China; CEU: Utah Residents with Northern and Western European ancestry; TSI: Toscani in Italy.

**Table S2: Correlations of genetic diversity measures with latitude, longitude and distances from glacial refugia.**

| All 17 populations | | latitude (p) | longitude (p) | km from FrancoCantabrian refugium (p) | km from NearEast refugium (p) |
|---|---|---|---|---|---|
| S/usable loci | Pearson | -0.748 (0.001) | 0.43 (0.085) | 0.378 (0.134) | -0.68 (0.003) |
| | Spearman | -0.675 (0.003) | 0.456 (0.066) | 0.362 (0.153) | -0.587 (0.013) |
| nd | Pearson | -0.566 (0.018) | 0.407 (0.105) | 0.424 (0.09) | -0.575 (0.016) |
| | Spearman | -0.491 (0.045) | 0.477 (0.053) | 0.522 (0.031) | -0.5 (0.041) |

| all except saami, palestinian, turkish | | latitude (p) | longitude (p) | km from FrancoCantabrian refugium (p) | km from NearEast refugium (p) |
|---|---|---|---|---|---|
| S/usable loci | Pearson | -0.32 (0.264) | 0.507 (0.064) | 0.455 (0.102) | -0.474 (0.087) |
| | Spearman | -0.416 (0.139) | 0.49 (0.075) | 0.383 (0.176) | -0.405 (0.151) |
| nd | Pearson | 0.024 (0.935) | 0.52 (0.057) | 0.697 (0.006) | -0.305 (0.289) |
| | Spearman | -0.068 (0.817) | 0.531 (0.051) | 0.678 (0.008) | -0.253 (0.383) |

S: number of polymorphic sites; nd: nucleotide diversity; p: p-value - blue color highlights significant (<0.05) values.
Analysis is of the coding region only. Correlation tests were run in R with the function cor.test of the package stats.
Anamur, Turkey (36.1°, 32.8°) and Fleurac, France (45.0°, 1.0°) were taken as proxies for the centres of the Near-East and Franco-Cantabrian refugia respectively.
Distances account for geographical barriers, and were estimated using the land transport distance tool at www.freemaptools.com.

**Table S3. Diversity parameters for the 17 populations for complete mtDNA sequences.**

| pop | N | k | S | # usable loci | S/usable loci | HD (sd) | MNPD (sd) | nd (sd) | D (p-value) | FS (p-value) |
|---|---|---|---|---|---|---|---|---|---|---|
| bas | 20 | 18 | 119 | 16381 | 0.0073 | 0.989 (0.019) | 18.421 (8.528) | 0.0011 (0.0006) | ***-1.858 (0.015)*** | -3.742 (0.077) |
| bav | 20 | 20 | 214 | 16564 | 0.0129 | 1 (0.016) | 33.858 (15.406) | 0.0020 (0.0010) | ***-1.823 (0.017)*** | ***-4.758 (0.018)*** |
| CEU | 20 | 20 | 155 | 16561 | 0.0094 | 1 (0.016) | 28.089 (12.836) | 0.0017 (0.0009) | ***-1.478 (0.047)*** | ***-5.582 (0.01)*** |
| den | 20 | 17 | 139 | 16403 | 0.0085 | 0.984 (0.020) | 29.521 (13.474) | 0.0018 (0.0009) | -1.019 (0.175) | -0.873 (0.325) |
| eng | 20 | 20 | 168 | 16562 | 0.0101 | 1 (0.016) | 26.011 (11.910) | 0.0016 (0.0008) | ***-1.868 (0.018)*** | ***-5.953 (0.013)*** |
| fri | 20 | 19 | 164 | 16197 | 0.0101 | 0.995 (0.018) | 25.353 (11.617) | 0.0016 (0.0008) | ***-1.871 (0.021)*** | -3.925 (0.056) |
| gre | 20 | 20 | 206 | 16128 | 0.0128 | 1 (0.016) | 31.953 (14.557) | 0.0020 (0.0010) | ***-1.868 (0.014)*** | ***-5.002 (0.015)*** |
| hun | 20 | 20 | 176 | 16352 | 0.0108 | 1 (0.016) | 27.605 (12.621) | 0.0017 (0.0009) | ***-1.839 (0.015)*** | ***-5.665 (0.013)*** |
| ire | 20 | 20 | 171 | 15995 | 0.0107 | 1 (0.016) | 29.895 (13.640) | 0.0019 (0.0010) | ***-1.574 (0.036)*** | -5.295 (0.022) |
| nor | 20 | 18 | 164 | 16401 | 0.0100 | 0.989 (0.019) | 31.642 (14.419) | 0.0019 (0.0010) | -1.307 (0.081) | -1.716 (0.178) |
| ork | 20 | 17 | 163 | 16559 | 0.0098 | 0.984 (0.021) | 30.942 (14.107) | 0.0019 (0.0010) | -1.353 (0.069) | -0.734 (0.347) |
| pal | 20 | 20 | 270 | 16563 | 0.0163 | 1 (0.016) | 38.168 (17.326) | 0.0023 (0.0012) | ***-2.076 (0.001)*** | -4.283 (0.039) |
| saa | 20 | 11 | 62 | 16255 | 0.0038 | 0.916 (0.041) | 15.0948 (7.046) | 0.0009 (0.0005) | -0.553 (0.334) | 2.151 (0.839) |
| ser | 20 | 20 | 165 | 16128 | 0.0102 | 1 (0.016) | 28.921 (13.207) | 0.0018 (0.0010) | ***-1.567 (0.037)*** | ***-5.446 (0.011)*** |
| spa | 20 | 20 | 170 | 16499 | 0.0103 | 1 (0.016) | 28.000 (12.797) | 0.0017 (0.0009) | ***-1.723 (0.026)*** | ***-5.597 (0.014)*** |
| TSI | 20 | 20 | 187 | 16560 | 0.0113 | 1 (0.016) | 28.626 (13.075) | 0.0017 (0.0009) | ***-1.89584 (0.008)*** | ***-5.494 (0.015)*** |
| tur | 20 | 20 | 244 | 16508 | 0.0148 | 1 (0.016) | 36.042 (16.379) | 0.0022 (0.0011) | ***-1.9802 (0.012)*** | -4.505 (0.031) |

pop: population; *N*: number of individuals; k: number of haplotypes; *S*: number of polymorphic sites; HD: haplotype diversity; MNPD: mean number of pairwise differences; nd: nucleotide diversity; *D*: Tajima's *D* (bold italic values are significant); FS: Fu's FS (bold italic values are significant).

**Table S4. DNA sequencing information**

| Dataset | N | Type of experiment | Library preparation | Read length | Platform |
|---|---|---|---|---|---|
| Illumina HiSeq | 201 | off-target data resulting from a sequence capture experiment (see Hallast et al. (2015) and Batini et al. (2015)). | Illumina Paired-End Sequencing Library kit (Version 1.3) | PE 100-bp | Illumina HiSeq 2000 |
| Ion Torrent PGM | 68 | Amplicon-seq<br>two overlapping 8/8.5-kb fragments amplified with primers (5´-3´):<br>F1-L802 [AAACGCTTAGCCTAGCCACA]<br>R1-H9369 [ACATCGCGCCATCATTGGT]<br>F2-L9338 [CCACTCCATAACGCTCCTCA]<br>R2- H814 [TTGCTAAAGGTTAATCACTGCTG] | Ion Xpress™ Template 200 Kit (Life Technologies) | SE 200-bp | Ion Torrent PGM [PGM™ Sequencing 200 Kit (Life Technologies) and Ion 314 chip] |
| Illumina MiSeq | 111 | Amplicon-seq<br>two overlapping 8.5/9-kb fragments amplified with primers (5´-3´):<br>F1-L802 (see above)<br>R1-H9369 (see above)<br>F2-L8874 [AGCGGGCACAGTGATTATAGG]<br>R2-H1290 [AGCCTTCATCAGGGTTTGCT] | Nextera XT DNA Library Preparation Kit | PE 150-bp | Illumina MiSeq [MiSeq Reagent Cartridge v2] |

N, number of individuals; PE, paired-end; SE, single-end

**References:**

Batini et al. 2015. Large-scale recent expansion of European patrilineages shown by population resequencing. Nature Comms 6: 7152.

Hallast et al. 2015. The Y-chromosome tree bursts into leaf: 13,000 high-confidence SNPs covering the majority of known clades. Mol Biol Evol 32: 661-673.

## Table S5: Software tools and parameters used in NGS data analysis.

| Function | Tool | Package | Parameters |
|---|---|---|---|
| Quality control | FastQC | | |
| Map reads | mem | bwa v0.7.5 | -R '@RG\tID:\tPL:\tLB:\tPU:\tSM:' rCRS |
| | TMAP software implemented in the Ion Alignment plugin 3.2.1 [*for IonTorrent PGM dataset only*] | | |
| | BAM files extracted from final alignments as described in Hallast et al. (2015) and Batini et al. (2015). | | |
| Pipeline below applied to all three datasets. | | | |
| Local realignment | RealignerTargetCreator | GATK v3.3.0 | -R rCRS[GenBank: J01415.2] |
| | IndelRealigner | | -R rCRS -targetIntervals file_made_by_RealignerTargetCreator |
| Duplicate marking | MarkDuplicates | Picard v1.84 | N/A |
| Base quality score recalibration | BaseRecalibrator | GATK v3.3.0 | -R rCRS -knownSites dbsnp138.b37 [mtDNA_only] (plus ReadGroupCovariate and QualityScoreCovariate by default) |
| | PrintReads | | -BQSR file_made_by_BaseRecalibrator |
| Coverage calculation | DepthOfCoverage | GATK 3.3.0 | -mbq 20 -mmq 50 |
| SNP calling | mpileup | SAMtools v1.1 | samtools mpileup -u -Q 20 -q 50 -I -g --output-tags DP,DV,DPR,INFO/DPR,SP -O -s -f rCRS/GRCh37-l analysed_coordinate_file |
| | bcftools call | BCFtools v1.1 | -m -S |
| Filtering | Vcftools v0.1.11 and Perl scripts | | Minimum DP 1 (HiSeq), 2 (MiSeq, IonTorrent) |

**References:**

Batini et al. 2015. Large-scale recent expansion of European patrilineages shown by population resequencing. Nature Comms 6: 7152.
Hallast et al. 2015. The Y-chromosome tree bursts into leaf: 13,000 high-confidence SNPs covering the majority of known clades. Mol Biol Evol 32: 661-673.

**Table S6: Variant validation summary.**

| Comparison | No. of overlapping samples | No. of overlapping sites | No. of calls compared | No of. FP/FN calls (%) |
|---|---|---|---|---|
| Our data (Illumina) vs HapMap Phase 1 sequence data | 76 | 879 | 60627 | 8 (0.013%)/ 1 (0.002%) |
| Our data (Illumina) vs validation sequence data (IonTorrent) | 10 | 16414 | 164002 | 0 (0%)/0 (0%) |
| Our data (IonTorrent) vs AffySNP6.0 PoBI mtDNA SNP data[a] | 6 | 407 | 2399 | 26 (1.084%)/ 6 (0.250%) |
| Our data (Illumina) vs AffySNP6.0 PoBI mtDNA SNP data[a] | 25 | 399 | 9825 | 81 (0.824%)/ 34 (0.346%) |

FP: false positive; FN: false negative; [a]: unpublished mtDNA SNP data associated with: Leslie S, Winney B, Hellenthal G, Davison D, Boumertit A, Day T, Hutnik K, Royrvik EC, Cunliffe B, Wellcome Trust Case Control C, International Multiple Sclerosis Genetics C, Lawson DJ, Falush D, Freeman C, Pirinen M, Myers S, Robinson M, Donnelly P, Bodmer W (2015) The fine-scale genetic structure of the British population. *Nature* **519**: 309-14.

## Table S7: List of possible 'phantom' mutations.

**Mutations recurring on the same haplogroup background and in the same population**[a]

| Position | No. of individuals | Samples | Soares Score |
|---|---|---|---|
| 309T | 2 | S132 [U2e3a]; S94 [U2e3a] | 0 |
| 1850C | 10 | utsa1 [U5b1b1a]; utsa13 [U5b1b1a]; utsa16 [U5b1b1a]; utsa18 [U5b1b1a]; utsa19 [U5b1b1a]; utsa2 [U5b1b1a]; utsa20 [U5b1b1a]; utsa4 [U5b1b1a]; utsa7 [U5b1b1a]; utsa8 [U5b1b1a] | 1 |
| 3327G | 2 | utsa5 [U5b1b1a]; utsa6 [U5b1b1a] | 1 |
| 4056T | 2 | den176 [H11a]; den183 [H11a] | 0 |
| 4958G | 2 | utsa5 [U5b1b1a]; utsa6 [U5b1b1a] | 2 |
| 5496G | 2 | ORK010 [X2b8]; ORK583 [X2b8] | 0 |
| 8756C | 2 | den176 [H11a]; den183 [H11a] | 0 |
| 9468G | 2 | ORK010 [X2b8]; ORK583 [X2b8] | 1 |
| 10786C | 2 | N24 [T2b2b]; N7 [T2b2b] | 2 |
| 14004T | 2 | bas12 [H1]; bas3 [H1] | 0 |
| 14875T | 2 | den192 [J1c4]; den34 [J1c4] | 1 |

**Mutations recurring on different haplogroup backgrounds and in different populations**[b]

| Position | No. of individuals | Samples | Soares Score | Minimum Coverage |
|---|---|---|---|---|
| *associated with LHON in den sample* [c] | | | | |
| 3460A | 3 | den150 [K1a11]; den50-51 [T2f7]; den82-83 [T2f7] | 1 | |
| 11778A | 13 | den152 [H13a1a1a]; den158 [T2e1a]; den176 [H11a]; den183 [H11a]; den186 [U8a1b]; den189 [H13a1a1a]; den190 [H10e]; den191 [K1a4a1]; den192 [J1c4]; den207 [H1c1]; den30 [T1a1k]; den34 [J1c4]; den36 [K1c1b] | 1 | |
| *sequenced on different platforms* [d] | | | | |
| 372C | 2 | NA19098 [L3b1a+152]; TK9 [R0a2] | 0 | >100X |
| 1494T | 2 | OXF023 [V]; fri1938 [U5a2c3a] | 1 | 85X |
| 2010C | 2 | 4918 [T2b]; SP2 [U5a1i] | 1 | 35X |
| 8659G | 2 | NA18632 [A15]; fri1938 [U5a2c3a] | 2 | 34X |
| 8772C | 2 | TK13 [T2a1b]; bav13 [U7b] | 2 | 11X |
| 9101C | 2 | GR99-4 [K1a]; HU3 [U6a5] | 2 | 13X |
| 9389G | 2 | ORK552 [T1a1]; SE3 [H7g] | 2 | 75X |
| 10792G | 2 | NA07357 [U5a1c2a]; bav19 [U4c1a] | 1 | 56X |
| 10975T | 2 | S0059 [T2b3b]; bas16 [T2e] | 0 | 10X |
| *sequenced on the same platform* [e] | | | | |
| 5318T | 2 | NA18609 [F2c2]; bas18 [H4a1a1a1a] | 0 | 24X |
| 12425G | 2 | TK7 [J1d3b]; bav40 [HV0] | 2 | >2000X |
| 15804C | 2 | NA20806 [H13b1b]; bas17 [X2c1] | 0 | 18X |
| 16220C | 2 | ORK008 [J1c2l]; SE20 [T2b] | 1 | 17X |

***Footnotes:***

**a**: Presence on same haplogroup and in same population implies that these are real variants, previously undetected.

**b**: Presence on different haplogroups and in different populations implies that these variants could in principle be artefactual ('phantom').

**c**: The Danish sample is known to include carriers of Leber Hereditary Optic Neuropathy (LHON), a mitochondrial disorder. These recognized LHON causative variants have been excluded from all analyses.

**d**: Unlikely to be platform-specific artefacts; also, minimum coverage across this set of variants is 10X, increasing our confidence that these are 'real' variants.

**e**: In principle these variants could be platform-specific artefacts; however, visual inspection and minimum coverage 17X does not suggest these are 'phantom' variants, as the alternative allele is observed in all aligned reads.
